# Comparison of the Accuracy and Completeness of Records of Serious Vascular Events in Routinely Collected Data vs Clinical Trial–Adjudicated Direct Follow-up Data in the UK: Secondary Analysis of the ASCEND Randomized Clinical Trial

**DOI:** 10.1001/jamanetworkopen.2021.39748

**Published:** 2021-12-28

**Authors:** Charlie Harper, Marion Mafham, William Herrington, Natalie Staplin, William Stevens, Karl Wallendszus, Richard Haynes, Martin J. Landray, Sarah Parish, Louise Bowman, Jane Armitage

**Affiliations:** 1 Medical Research Council Population Health Research Unit at the University of Oxford, Nuffield Department of Population Health (NDPH), University of Oxford, Oxford, United Kingdom; 2 Clinical Trial Service Unit and Epidemiological Studies Unit, NDPH, University of Oxford, Oxford, United Kingdom; 3 Big Data Institute, Li Ka Shing Centre for Health Information and Discovery, NDPH, University of Oxford, Oxford, United Kingdom

## Abstract

**Importance:**

Routinely collected data could substantially decrease the cost of conducting trials.

**Objective:**

To assess the accuracy and completeness of UK routine data for ascertaining serious vascular events (SVEs) compared with adjudicated follow-up data.

**Design, Setting, and Participants:**

This was a secondary analysis of a randomized clinical trial. From June 24, 2005, to July 28, 2011, the ASCEND (A Study of Cardiovascular Events in Diabetes) primary prevention trial used mail-based methods to randomize people with diabetes without evidence of atherosclerotic vascular disease using a 2 × 2 factorial design to aspirin and/or ω-fatty acids vs matching placebo in the UK. Direct participant mail-based follow-up was the main source of outcome data, with more than 90% of the primary outcome events undergoing adjudication. Follow-up was completed on July 31, 2017. In parallel, more than 99% of participants were linked to routinely collected hospital admission and death registry data (ie, routine data), enabling post hoc randomized comparisons of different sources of outcome data (conducted from September 1, 2018, to October 1, 2021).

**Interventions:**

Random allocation to 100 mg of aspirin once daily vs matching placebo and separately to 1 g of ω-3 fatty acids once daily vs placebo.

**Main Outcomes and Measures:**

The primary outcome consisted of SVEs (a composite of nonfatal myocardial infarction, ischemic stroke, transient ischemic attack [TIA], or vascular death, excluding hemorrhagic stroke).

**Results:**

A total of 15 480 participants were randomized (mean [SD] age, 63 [9] years; 9684 [62.6%] men) and followed up for a mean (SD) of 7.4 (1.8) years. For SVEs, agreement between adjudicated direct follow-up and routine data sources was strong (1401 vs 1127 events; κ = 0.78 [95% CI, 0.76-0.80]; sensitivity, 72.0% [95% CI, 69.7%-74.4%]; specificity, 99.2% [95% CI, 99.0%-99.3%]), and sensitivity improved for SVEs excluding transient ischemic attack (1129 vs 1026 events; sensitivity, 80.6% [95% CI, 78.3%-82.9%]). Rate ratios for the aspirin-randomized comparison for adjudicated direct follow-up vs follow-up solely through routine data alone were 0.88 (95% CI, 0.79-0.97) vs 0.91 (95% CI, 0.81-1.02) for the primary outcome and 0.92 (95% CI, 0.82-1.03) vs 0.91 (95% CI, 0.80-1.02) for SVEs excluding TIA. Results were similar for the ω-3 fatty acid comparison, and adjudication did not seem to markedly change rate ratios.

**Conclusions and Relevance:**

Post hoc analyses of the ASCEND trial suggest that routinely collected hospital admission and death registry data in the UK could be used as the sole method of follow-up for myocardial infarction, ischemic stroke resulting in hospitalization, vascular death, and arterial revascularization in primary prevention cardiovascular trials, without the need for verification by clinical adjudication.

## Key Points

**Question:**

Are routinely collected data sufficiently accurate and complete to be a trial’s sole follow-up method for serious vascular events (SVEs)?

**Findings:**

In post hoc analyses of ASCEND (A Study of Cardiovascular Events in Diabetes), a trial including 15 480 UK residents with diabetes, routine data showed strong agreement for SVEs compared with adjudicated follow-up. On rerun randomized analyses, follow-up using routine data provided similar estimates of effect sizes on SVEs to adjudicated follow-up for both aspirin vs placebo and ω-3 fatty acids vs placebo comparisons.

**Meaning:**

These routinely collected UK data potentially provide a sufficiently reliable method of ascertaining fatal and hospitalized SVEs, without the need for verification by clinical adjudication.

## Teaser

This post hoc secondary analysis of a randomized clinical trial assesses whether routinely collected health data or streamlined mail-based follow-up data without adjudication are sufficiently accurate and complete to be the sole source of estimates of serious vascular events in patients with diabetes.

## Introduction

Large randomized trials in cardiovascular disease have provided reliable evidence for interventions whose widespread use has contributed to the secular declines in mortality due to cardiovascular disease.^1,2^ However, increasing costs of conducting trials threaten our ability to generate new randomized evidence.^3,4^ Observational analyses in routinely collected health care (ie, real-world) data are increasingly used to make inferences about the safety and efficacy of interventions, but such nonrandomized analyses cannot fully account for confounding and are unreliable.^5,6^ More appropriately, routinely collected data could be used to streamline trial follow-up conduct and design by offering a cost-efficient method to ascertain outcomes.^7,8^

The National Health Service (NHS) in the UK has long-established, routinely collected national level mortality and hospital admission records, including Hospital Episode Statistics^9^ in England, with equivalents in Wales^10^ and Scotland.^11^ These records cover all NHS hospitals and have been collected since the 1990s using standardized clinical coding practices,^12,13,14^ but have rarely been adopted into trial designs.^15,16,17,18^ This is perhaps owing to the limited evidence of their accuracy and completeness for specific outcomes and delays in receiving the data.^19^

ASCEND (A Study of Cardiovascular Events in Diabetes)^20,21,22^ is a large double-blind placebo-controlled trial including individuals with diabetes but no evidence of atherosclerotic vascular disease at recruitment (ie, a primary prevention population) conducted in the UK from June 24, 2005, to July 31, 2017. A total of 15 480 participants were randomized and followed up by regular mail-based questionnaires, and reports of possible serious vascular events (SVEs, a composite of nonfatal myocardial infarction, ischemic stroke, transient ischemic attack [TIA], or vascular death [excluding intracranial hemorrhage]) or revascularizations underwent clinical adjudication. Participants were also linked to national death and cancer registries and routinely collected hospital admission data. We aimed to assess whether such routinely collected data are sufficiently accurate and complete to be the sole source of serious vascular outcome ascertainment. Analyses compared the ASCEND results using adjudicated direct participant follow-up with 2 alternative hypothetical scenarios: (1) following up participants solely through routinely collected health care data systems and (2) performing streamlined mail-based follow-up without adjudication.

## Methods

### ASCEND Trial

**Figure 1.**
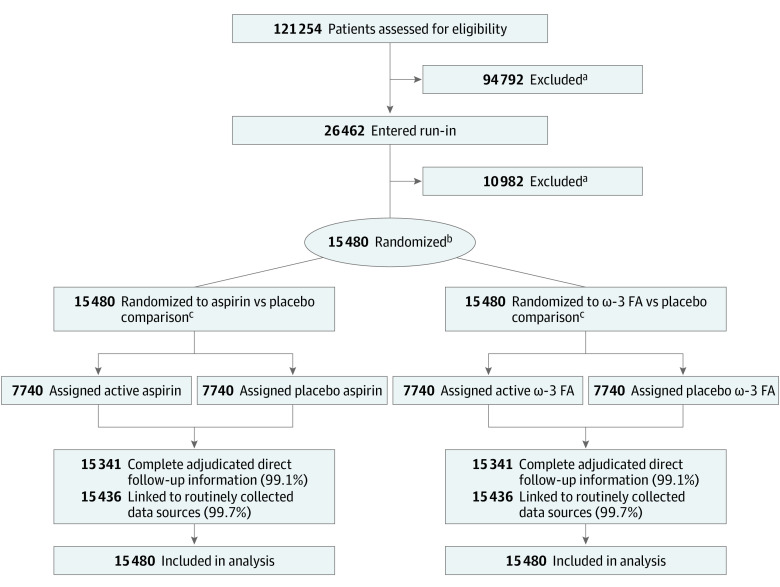
Flow Diagram of Post Hoc Analyses in ASCEND (A Study of Cardiovascular Events in Diabetes) FA indicates fatty acids. ^a^A complete breakdown of exclusions can be found in ASCEND’s main publications. ^b^Randomization used a 2 × 2 factorial design. ^c^All 15 480 participants were included in both the aspirin and ω-3 FA comparisons.

Post hoc analyses were conducted using the ASCEND trial, with design and findings having been reported previously (Figure 1) (a complete copy of the trial protocol is provided in Supplement 1).^20,21,22^ Briefly, from June 24, 2005, to July 28, 2011, 15 480 UK participants were randomized using a 2 × 2 factorial design to low-dose aspirin (100 mg once per day) vs placebo and separately to ω-3 fatty acids (1 g once per day) vs placebo. The primary efficacy assessment was time to first SVE (a composite of nonfatal myocardial infarction, presumed ischemic stroke or TIA, and vascular death [excluding intracranial hemorrhage]). A secondary assessment was time to first SVE or any arterial revascularization. The mean (SD) follow-up was 7.4 (1.8) years. The protocol was approved by the North West Multicenter Research Ethics Committee, and participants provided written informed consent. This study followed the Consolidated Standards of Reporting Trials (CONSORT) reporting guideline.

### Direct Participant Follow-up and Adjudication

The principal method of direct participant recruitment and follow-up was by mailed questionnaire (traditional and, in later years of the follow-up period for some participants, electronic) every 6 months until the end of the trial, termed the *preadjudicated direct follow-up* (eFigure 1 in Supplement 2). The primary and secondary outcomes and whether participants were hospitalized or not were adjudicated by study clinicians who were blinded to treatment allocation. For nonfatal vascular outcomes, the coordinating center requested documentation (eg, hospital discharge summaries) from primary care. For the 1076 nonfatal SVEs reported in the primary results, 514 events (47.8%) were ascertained via participant mail-based follow-up; 260 (24.2%), via telephone contact with the participant; 255 (23.7%), from contact with their general practitioner or hospital physician; 28 (2.6%), via other means; and 19 (1.8%), via routine hospital admission data when (in a small number of cases) participants were lost to follow-up. For fatal outcomes, the primary source of information was the Office for National Statistics death certification data, which included deaths occurring both within and out of hospital. The reported primary and subsidiary causes of death were then reviewed by study clinicians (including M.M.) along with available hospital admissions data and any information from the trial questionnaires. The published results were based on all unrefuted reports of serious adverse events (ie, a reported event for which supporting documentation could not be obtained was counted in the analysis). More than 90% of the SVEs included in the analysis were verified by adjudicators. These outcome data from adjudicated follow-up presented in the ASCEND primary publications^21,22^ are termed *adjudicated direct follow-up* in this report (eFigure 1 in Supplement 2). Details of the adjudication definitions (including how deaths were ascribed to their underlying cause using information from review of other clinical information alongside death certificates) are provided in Appendix A of Bowman et al.^21^

### Routinely Collected Death Records and Hospital Admission Data

Written informed consent was obtained from participants to allow access to their routinely collected data. During follow-up, death records were obtained from the Office for National Statistics via NHS Digital for England/Wales^23^ and National Health Scotland Central Register.^24^ These data included date of death and underlying and other contributing causes of death. Participants were also linked to their routinely collected hospital admission records; these data were obtained from NHS Digital (Hospital Episode Statistics Admitted Patient Care)^9^ for England, Public Health Scotland (Scottish Morbidity Records 01),^11^ and Welsh SAIL Databank (Patient Episode Database for Wales).^10^ Information used for these analyses included primary and any secondary diagnoses, any operations and procedures, and admission dates. Linkage between the trial participants and routine data sets was performed by the national registries but was not possible for 44 participants (0.3%) residing in Northern Ireland, among whom only 1 SVE occurred during follow-up. At the time of this study, participants were not linked to their primary care general practice data.

All deaths in the UK are included in the mortality registry, irrespective of location of death, and medically certified by a physician or pathologist. For routine data analyses, deaths with vascular disease as the underlying cause were identified from the register, which uses an automated algorithm based on principles established in the *International Statistical Classification of Diseases and Related Health Problems, Tenth Revision* (*ICD-10*) (categorization is provided in eTable 1 in Supplement 2). Nonfatal SVEs were identified in hospital admission data using *ICD-10* diagnosis codes in any diagnostic position (ie, primary or secondary). The event date was assumed to be the admission date because there was no diagnosis date in the available routine data. Any arterial revascularization procedures (including dates) were identified using procedure codes from the *Office of Population Censuses Surveys Classification of Surgical Operations and Procedures, Version 4*. Myocardial infarctions and ischemic strokes were assumed to be nonfatal unless they were within 30 days of such deaths ascribed to such causes. Events identified through routinely collected data did not undergo clinical adjudication and are hereinafter termed *routine data* (eFigure 1 in Supplement 2).

### Statistical Analysis

Analyses included only those events that occurred from randomization to the date of death or censoring, except for participants from Northern Ireland, where routine follow-up data was censored at day zero. Outcomes identified using routine data were compared with those from adjudicated direct participant follow-up. For each outcome, participants were categorized as having an outcome in both data sets (ie, routine data identified an outcome that had also been ascertained by adjudicated direct follow-up); an outcome in routine data only (ie, not ascertained in adjudicated direct follow-up); an outcome in adjudicated direct follow-up alone (ie, outcomes not recorded in routine data); or no such outcome in either data set. Levels of agreement between the 2 data sources were assessed using sensitivity and specificity with 95% CIs.^25^ Sensitivity (ie, completeness) was calculated by dividing the number of participants with an outcome in both data sets by the total number of participants with an outcome reported via adjudicated follow-up. Specificity (ie, accuracy) was calculated by dividing no such outcome in either data set by the number of participants with no such outcome reported via adjudicated follow-up. Overall levels of agreement were estimated using the κ statistic^26^ with 95% CIs and interpreted using an established approach^27^ where 0.01 to 0.20 represents slight agreement; 0.21 to 0.40, fair agreement; 0.41 to 0.60, moderate agreement; 0.61 to 0.80, strong agreement; and 0.80 to 0.99, very strong agreement.

Differences in κ statistics between subgroups were assessed using heterogeneity testing,^26,28^ including analyses by participants’ mean age (<63 vs ≥63 years), sex (male vs female), vascular risk score (low vs medium vs high [see ASCEND data analysis plan^20^]), and country of residence (England vs other UK country). When there was agreement between the 2 sources of outcome data (ie, outcome in both data sets), the event dates were compared. Differences were presented as an exact match at 1 to 7 days, 8 to 30 days, 31 to 90 days, 91 to 180 days, and more than 180 days. Where an SVE was reported in adjudicated follow-up alone, routine data were searched to identify whether there was a corresponding hospitalization (within 90 days of the adjudicated event date) or death record for the same participant. A similar process was performed for events recorded in routine data only.

All randomized comparisons used standard log-rank methods^29,30^ following the intention-to-treat approach. Rate ratios (RRs) with 95% CIs are provided. The main randomized comparisons were those based on the adjudicated direct follow-up data vs those based on the alternative scenario that ASCEND had only used routine data to identify outcomes. Sensitivity analyses included restricting adjudicated follow-up to fatal and hospitalized events only (in which events where adjudicated hospitalization status was unknown were assumed to have not led to an admission) and analyses restricted to only using diagnoses in the primary position of the hospital admission record. For the main randomized comparisons, differences between the RRs for adjudicated follow-up vs routine data were calculated with 95% CIs derived using bootstrap methods. Secondary analyses included randomized comparisons using outcomes derived from preadjudicated direct follow-up. Analyses were conducted from September 1, 2018, to October 1, 2021, using SAS, version 9.4 (SAS Institute Inc), and R, version 4.1.1 (R Project for Statistical Computing). Two-sided *P* < .05 indicated statistical significance.

## Results

### Baseline Characteristics

A total of 15 480 participants were included in this study; mean (SD) age was 63 (9) years; 9684 (62.6%) were men and 5796 (37.4%) were women. Hypertension was reported in 9533 participants (61.6%). The median duration of diabetes was 7 (IQR, 3-13) years, and 13 960 participants (90.2%) resided in England (eTable 2 in Supplement 2).

### Accuracy and Completeness of Routine Data

There were 1401 unrefuted SVEs within adjudicated direct follow-up, of which 1009 were also identified in the routine data, so sensitivity of routine data was 72.0% (95% CI, 69.7%-74.4%) (Table 1). One hundred eighteen events were recorded in the routine data but not confirmed in adjudicated direct follow-up, and 13 961 participants had no event in either data set; hence, specificity of routine data was 99.2% (95% CI, 99.0%-99.3%). Overall agreement between routine data and adjudicated follow-up for any SVE was strong (1401 vs 1127 events; κ = 0.78 [95% CI, 0.76-0.80]), and sensitivity improved for SVEs excluding TIA (1129 vs 1026 events; sensitivity, 80.6% [95% CI, 78.3%-82.9%]).

**Table 1.** Agreement of Routine Data vs Adjudicated Direct Follow-up Data

Outcome	Participants, No. (%) (N = 15 480)				Sensitivity, % (95% CI)	Specificity, % (95% CI)	κ (95% CI)
	Outcome in both data sets	Outcome in routine data only	Outcome in adjudicated follow-up alone	No such outcome in either data set			
Nonfatal MI	304 (2.0)	79 (0.5)	82 (0.5)	15 015 (97.0)	78.8 (74.7-82.8)	99.5 (99.4-99.6)	0.79 (0.75-0.82)
Nonfatal presumed ischemic stroke	288 (1.9)	65 (0.4)	143 (0.9)	14 984 (96.8)	66.8 (62.4-71.3)	99.6 (99.5-99.7)	0.73 (0.69-0.76)
Vascular death excluding ICH	365 (2.4)	18 (0.1)	49 (0.3)	15 048 (97.2)	88.2 (85.1-91.3)	99.9 (99.8-99.9)	0.91 (0.89-0.93)
Any serious vascular event excluding TIA	910 (5.9)	116 (0.7)	219 (1.4)	14 235 (92.0)	80.6 (78.3-82.9)	99.2 (99.0-99.3)	0.83 (0.82-0.85)
TIA	109 (0.7)	30 (0.2)	256 (1.7)	15 085 (97.4)	29.9 (25.2-34.6)	99.8 (99.7-99.9)	0.43 (0.36-0.49)
Any serious vascular event including TIA	1009 (6.5)	118 (0.8)	392 (2.5)	13 961 (90.2)	72.0 (69.7-74.4)	99.2 (99.0-99.3)	0.78 (0.76-0.80)
Any arterial revascularization	685 (4.4)	51 (0.3)	39 (0.3)	14 705 (95.0)	94.6 (93.0-96.3)	99.7 (99.6-99.7)	0.94 (0.92-0.95)
Any SVE or revascularization	1404 (9.1)	127 (0.8)	365 (2.4)	13 584 (87.8)	79.4 (77.5-81.3)	99.1 (98.9-99.2)	0.83 (0.82-0.85)

Abbreviations: ICH, intracranial hemorrhage; MI, myocardial infarction; SVE, serious vascular event; TIA, transient ischemic attack.

Specificity of routine data was very strong (>99%) for all components of any SVE and for arterial revascularizations, but sensitivity varied. Sensitivity was highest for any arterial revascularization at 94.6% (685 of 724 events; 95% CI, 93.0%-96.3%) and vascular death at 88.2% (365 of 414 events; 95% CI, 85.1%-91.3%) (Table 1). For myocardial infarction, sensitivity was 78.8% (304 of 386 events; 95% CI, 74.7%-82.8%); for nonfatal presumed ischemic stroke, it was lower (66.8% [288 of 431 events; 95% CI, 62.4%-71.3%]), and sensitivity was lowest for TIA (29.9% [109 of 365 events; 95% CI, 25.2%-34.6%]). Levels of agreement for any SVE were generally consistent by subgroups, except vascular risk score, where participants with a low score showed significantly lower agreement (κ = 0.71 [95% CI, 0.66-0.75]) compared with a medium score (κ = 0.79 [95% CI, 0.76-0.81]) or a high score (κ = 0.80 [95% CI, 0.77-0.84]) (eTable 3 in Supplement 2). Taking codes in any diagnosis position vs the primary position, the sensitivity improved appreciably without any compromise on the specificity (eTable 4 in Supplement 2). For the 1009 SVEs in which there was agreement between adjudicated direct follow-up and routine data, date of hospital admission was accurate to within 1 week for 857 events (84.9%) (eTable 5 in Supplement 2). Where there was disagreement between the 2 data sources for any SVE outcome, a full breakdown of the 188 events in routine data only and 392 in adjudicated follow-up alone can be found in eTables 6 and 7 in Supplement 2.

The sources of routine data used for these analyses were limited to capturing mostly fatal events and those resulting in hospitalization (hereinafter termed *hospitalized events*), and only 1099 of 1401 SVEs (78.4%) reported in adjudicated follow-up were recorded in the trial as involving hospitalization or death. When adjudicated follow-up was restricted to fatal and hospitalized events only, sensitivity of routine data was found to be higher (86.4% [95% CI, 84.4%-88.5%]) while maintaining strong specificity (98.8% [95% CI, 98.6%-98.9%]) (eTable 8 in Supplement 2). Of the adjudicated follow-up reported ischemic strokes and transient ischemic attacks, 306 of 431 (71.0%) and 120 of 365 (32.9%), respectively, led to hospitalization or death. When adjudicated follow-up was restricted to fatal and hospitalized events only, sensitivity of routine data was very strong for ischemic strokes (85.0% [260 of 306 events; 95% CI, 81.0%-89.0%]) and was moderate for transient ischemic attacks (59.2% [71 of 120 events; 95% CI, 50.4%-68.0%]).

### Randomized Comparisons Using Routine Data

**Figure 2.**
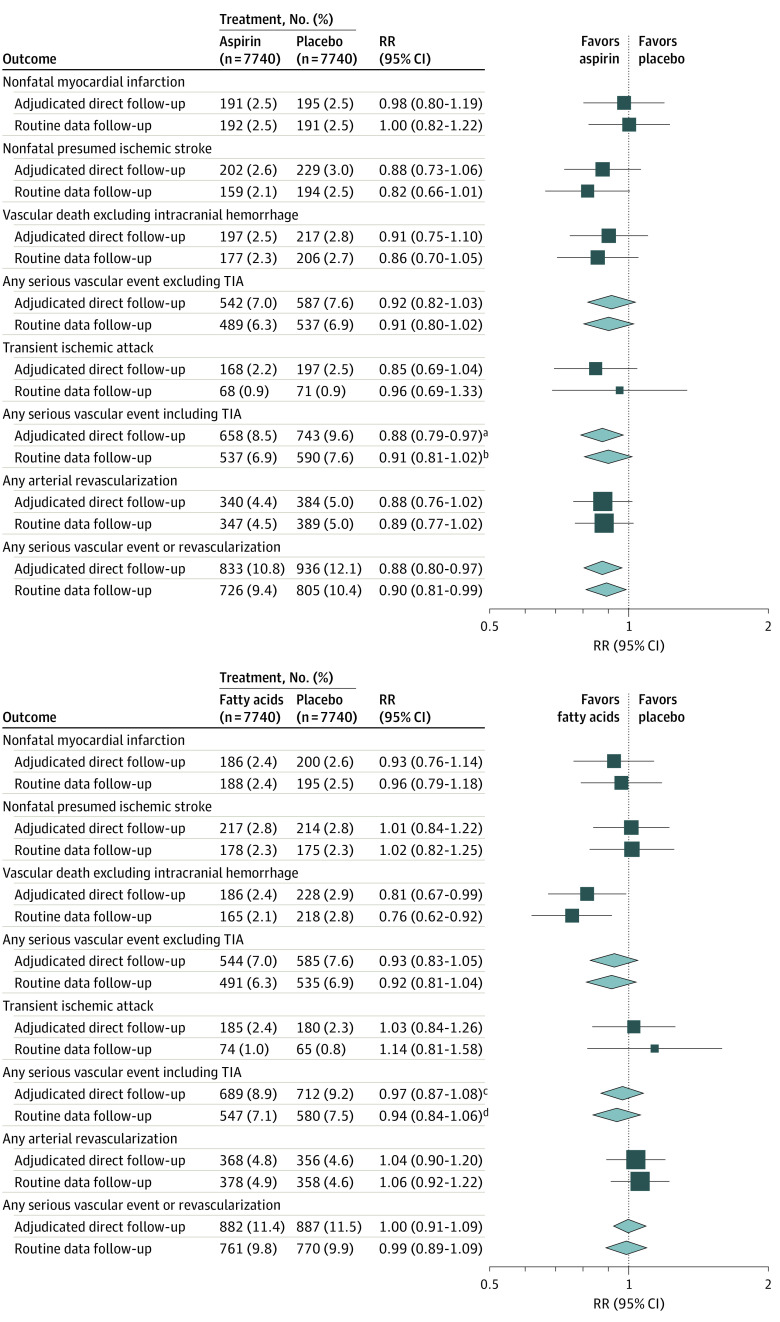
Rate Ratios for Serious Vascular Events Using Routine Data and Adjudicated Direct Follow-up Data Log-rank methods were used to calculate rate ratios (RRs) and 95% CIs. The size of the square for each RR is proportional to the amount of statistical information that was available; the horizontal lines represent 95% CIs. For composite outcomes, RRs and their corresponding 95% CIs are represented by diamonds. TIA indicates transient ischemic attack. ^a^*P* = .01. ^b^*P* = .10. ^c^*P* = .55. ^d^*P* = .32.

Overall, the randomized comparisons provided similar estimates of treatment effect size based on adjudicated direct follow-up for SVEs (658 [8.5%] for aspirin vs 743 [9.6%] for placebo; RR, 0.88 [95% CI, 0.79-0.97]) vs those using routine data alone (537 [6.9%] for aspirin vs 590 [7.6%] for placebo; RR, 0.91 [95% CI, 0.81-1.02]) (Figure 2). Rate ratios were almost identical for SVEs excluding TIA using adjudicated follow-up (542 [7.0%] for aspirin vs 587 [7.6%] for placebo; RR, 0.92 [95% CI, 0.82-1.03]) vs using routine data alone (489 [6.3%] for aspirin vs 537 [6.9%] for placebo; RR, 0.91 [95% CI, 0.80-1.02]). Similar findings were apparent for analyses of the effect size of ω-3 fatty acids vs placebo with SVEs using adjudicated follow-up (689 [8.9%] for ω-3 fatty acids vs 712 [9.2%] for placebo; RR, 0.97 [95% CI, 0.87-1.08]) vs using routine data alone (547 [7.1%] for ω-3 fatty acids vs 580 [7.5%] for placebo; RR, 0.94 [95% CI, 0.84-1.06]). For SVEs, the difference in estimated RRs between adjudicated direct follow-up and routine data was 0.03 (95% CI, −0.04 to 0.10) for the aspirin-randomized comparison and −0.03 (95% CI, −0.10 to 0.05) for the ω-3 fatty acids comparison (eTable 9 in Supplement 2). When adjudicated follow-up was restricted to fatal and hospitalized SVEs only, treatment effect sizes for both the aspirin (526 [6.8%] vs 573 [7.4%] for placebo; RR, 0.91 [95% CI, 0.81-1.03]) and ω-3 fatty acids (536 [6.9%] vs 563 [7.3%] for placebo; RR, 0.95 [95% CI, 0.85-1.07]) comparisons were almost identical to the above routine data follow-up results (eFigure 2 in Supplement 2).

### Adjudication

**Figure 3.**
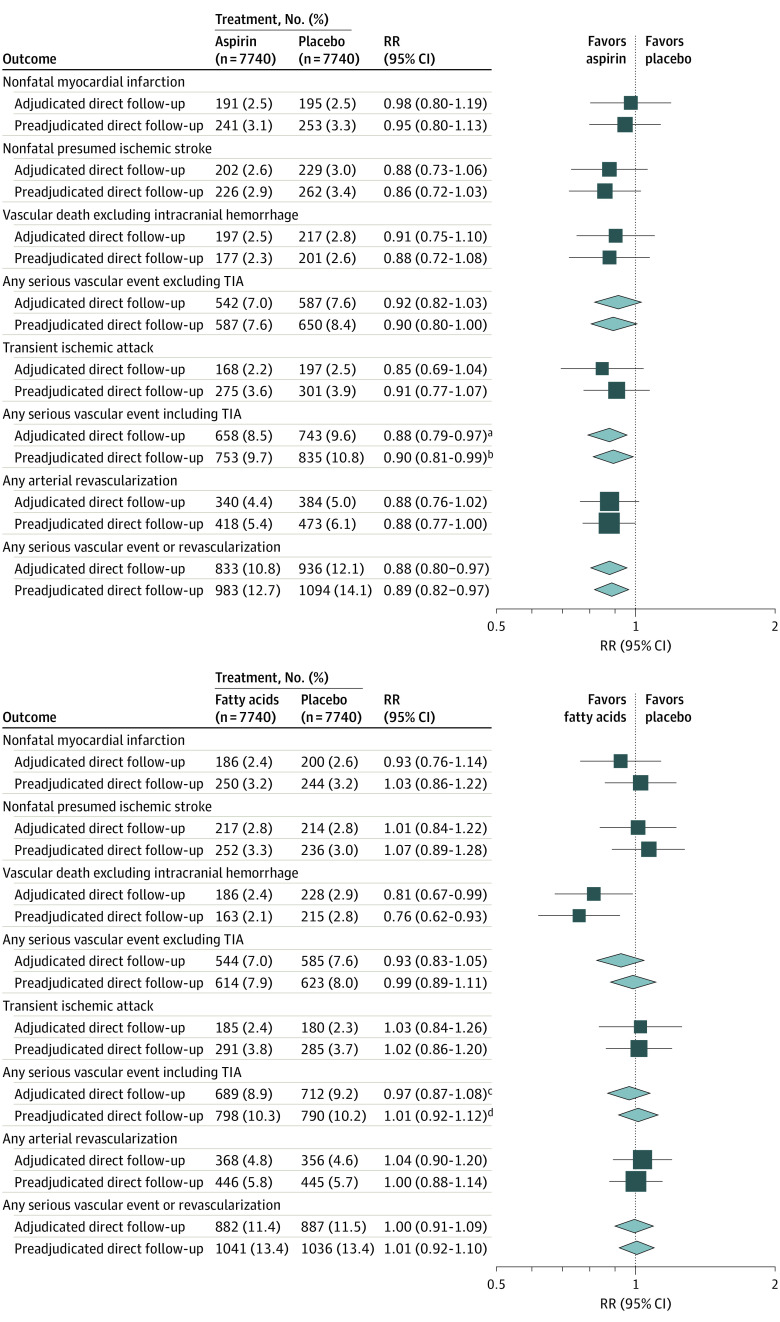
Rate Ratios for Serious Vascular Events Using Adjudicated and Preadjudicated Direct Follow-up Data Log-rank methods were used to calculate rate ratios (RRs) and 95% CIs. The size of the square for each RR is proportional to the amount of statistical information that was available; the horizontal lines represent 95% CIs. For composite outcomes, RRs and their corresponding 95% CIs are represented by diamonds. TIA indicates transient ischemic attack. ^a^*P* = .01. ^b^*P* = .03. ^c^*P* = .55. ^d^*P* = .79.

Adjudication verified approximately three-quarters of participant mail-based reported myocardial infarctions (363 of 494 [73.5%]) and presumed ischemic strokes (371 of 488 [76.0%]) and more than 80% of reported coronary revascularizations (521 of 638 [81.7%]) (Table 2). Of the 355 reported noncoronary revascularizations, only 189 (53.2%) were verified by adjudication. Reviewing 968 reports of hospitalized angina yielded only 86 nonfatal myocardial infarctions (8.9%), whereas review of 576 TIAs confirmed 338 (58.7%) and identified 110 (19.1%) presumed ischemic strokes. For randomized analyses, the relative effect sizes of treatment for SVEs remained largely unchanged for aspirin comparisons for adjudicated direct follow-up (658 [8.5%] vs 743 [9.6%] for placebo; RR, 0.88 [95% CI, 0.79-0.97) vs preadjudicated direct follow-up (753 [9.7%] vs 835 [10.8%] for placebo; RR, 0.90 [95% CI, 0.81-0.99]) and for ω-3 fatty acids comparisons for adjudicated follow-up (689 [8.9%] vs 712 [9.2%] for placebo; RR, 0.97 [95% CI, 0.87-1.08]) vs preadjudicated follow-up (798 [10.3%] vs 790 [10.2%] for placebo; RR, 1.01 [95% CI, 0.92-1.12]) (Figure 3).

**Table 2.** Agreement of Preadjudicated vs Adjudicated Direct Follow-up

Outcome before adjudication	Outcome after adjudication, No. (%)^a^									
	Nonfatal MI	Angina hospitalization	Coronary revascularization	Noncoronary revascularization	TIA	Presumed ischemic stroke	Hemorrhagic stroke	Subdural hemorrhage	Other^b^	All
Nonfatal MI	363 (73.5)	13 (2.6)	2 (0.4)	0	1 (0.2)	1 (0.2)	0	0	114 (23.1)	494 (100)
Angina hospitalization	86 (8.9)	285 (29.4)	9 (0.9)	0	1 (0.1)	0	0	0	587 (60.6)	968 (100)
Coronary revascularization	0	0	521 (81.7)	37 (5.8)	0	1 (0.2)	0	0	79 (12.4)	638 (100)
Noncoronary revascularization	0	0	22 (6.2)	189 (53.2)	0	0	0	0	144 (40.6)	355 (100)
TIA	0	0	0	0	338 (58.7)	110 (19.1)	4 (0.7)	2 (0.3)	122 (21.2)	576 (100)
Presumed ischemic stroke	0	0	0	1 (0.2)	38 (7.8)	371 (76.0)	7 (1.4)	3 (0.6)	68 (13.9)	488 (100)
Hemorrhagic stroke	0	0	0	0	0	1 (4.0)	21 (84.0)	2 (8.0)	1 (4.0)	25 (100)
Subdural hemorrhage	0	0	0	0	0	0	0	29 (93.5)	2 (6.5)	31 (100)
All, No.^c^	386	296	544	202	365	431	32	38	NA	NA

Abbreviations: MI, myocardial infarction; NA, not applicable; TIA, transient ischemic attack.

^a^ Percentages in parentheses are percentages of total number of ASCEND (A Study of Cardiovascular Events in Diabetes) participants with the outcome reported before adjudication. The total (for each row or column) counts only the first event that occurred for each participant.

^b^ Includes all events that were adjudicated to categories not represented by the other columns.

^c^ The sum of each column does not equal the total because participants may have had more than 1 event (eg, during the follow-up a participant may have had a nonfatal MI and hospitalization for angina).

## Discussion

In these post hoc analyses from the ASCEND trial, we found that routinely collected information about SVEs (a composite of nonfatal myocardial infarction, presumed ischemic stroke or TIA, and vascular death [excluding intracranial hemorrhage]) and arterial revascularizations based on hospitalization and national mortality records in the UK provided similar estimated treatment effect sizes to adjudicated follow-up for both the aspirin and ω-3 fatty acids randomized comparisons. Agreement between adjudicated follow-up and routine data was strong for these SVEs, with sensitivity substantially improved when adjudicated follow-up was restricted to fatal and hospitalized SVEs only. Furthermore, the treatment effect sizes were similar when mail-based preadjudicated direct participant follow-up reports were compared with adjudicated outcome data. Sensitivity of routine data to ascertain both nonfatal presumed ischemic strokes and particularly TIAs was lower than for vascular death and cardiac components of SVEs. This could be partly owing to approximately one-third of strokes being managed within outpatient stroke clinics in the UK.^31,32^ However, routine data appeared to identify some additional events potentially missed by direct participant follow-up. Therefore, although future trials could ascertain myocardial infarctions, hospitalized ischemic strokes, arterial revascularizations, and vascular deaths solely from routine data, if a wider range of cerebrovascular outcomes is prespecified to be recorded, this will require direct participant follow-up and/or additional data sets such as UK primary care data.

Our findings for nonfatal myocardial infarctions confirm the hypotheses raised by post hoc analyses of the WOSCOPS (West of Scotland Coronary Prevention Study) (1989-1995) linked to Scottish routine data.^33,34^ Using more modern hospital data from the UK, the ASCEND trial (2005-2017) suggests that myocardial infarction events are recorded even more completely than the earlier Scottish data (WOSCOPS vs ASCEND sensitivity for routine data compared with adjudicated follow-up: 230 of 428 [53.7%] vs 304 of 386 [78.8%]), with adjudication no longer being necessary. For nonfatal ischemic strokes, recent observations from Oxfordshire hospital admissions data also corroborate our findings: approximately two-fifths of ischemic strokes did not result in hospitalization, and of those that did, only approximately 70% were identifiable using Hospital Episode Statistics.^31^ The presented randomized findings from ASCEND in the UK also mirror findings from similar analyses performed using data from randomized trials linked to insurance claims data from the US^35^ and separately to public registers in Denmark.^36^

The point estimates in the randomized comparisons based solely on routine health care data in ASCEND show no evidence of being materially altered for the primary outcome of SVEs or components of that composite outcome based on adjudicated direct follow-up (ie, there was little evidence of bias, at least for these outcomes in this single trial) (eTable 9 in Supplement 2). However, the 22% reduction in the numbers of SVEs from 1401 identified by participant reporting that were then adjudicated vs 1127 SVEs identified through record linkage alone has implications for sample size calculations. If ASCEND had been designed to solely use routinely collected death and hospitalization data for follow-up, we estimate it would have been necessary for approximately 3500 more participants to be randomized to retain its intended power (if followed up for the same duration). However, the efficiency of using routine data arguably outweighs this disadvantage and enables low-cost long-term follow-up. Investigators and trial steering committees can pay particular attention to early event rates in trials reliant on routine data for their follow-up to ensure reassessment of design assumptions and recommend protocol modification, where necessary. Indeed, monitoring of event rates in ASCEND led to the important decision to expand the trial from its originally intended sample size of 10 000.^20^

The RRs for the analyses of the primary SVE outcome were similar when SVE outcomes were ascertained solely from mail-based questionnaire follow-up without adjudication. The presented analyses provide evidence that participant-reported SVEs are sufficiently reliable so that confirmation by clinician adjudicators is unnecessary for most vascular events. This is consistent with meta-analysis results from methodological studies demonstrating that adjudication made little difference to the observed RRs compared with vascular outcomes ascertained at research clinics.^37^ ASCEND differs from these other studies because it relied on participant reports from mailed questionnaires rather than the more resource-intensive clinic-based follow-up with trained research staff.

Depending on the nature of the trial, data on events need to be collected in a timely manner.^38^ If the only source of such information is routine data, it is important that these are received soon enough for relevant clinical decisions to be made. For example, pharmacovigilance reporting timelines may require alternative methods of rapid reporting of suspected serious adverse reactions (eg, 24-hour telephone services). However, information from routinely collected sources may be sufficiently timely for data monitoring committees’ serial reviews of randomized comparisons, despite outcomes not being recorded immediately. Current collaborative efforts in the UK between researchers and data providers through the recently founded Health Data Research UK^39^ mean that a greater amount of routinely collected health care data may be accessible to trialists within the time frames needed to monitor the safety of trial participants.^40,41^ In addition, in several non-UK countries, cardiovascular trials have been successfully embedded into routine health care data systems, such as TASTE (Thrombus Aspiration in Myocardial Infarction)^42^ in Sweden^43^ and ADAPTABLE (Aspirin Dosing: A Patient-Centric Trial Assessing Benefits and Long-term Effectiveness)^44^ in the US.^45^

### Limitations

This study has some limitations. First, ASCEND was a primary prevention population limiting generalizability to secondary prevention trials, in which a distinction must be made between an incident SVE and the recording of prerandomization diseases. Second, ASCEND participants have not been linked to their primary care records, which may have improved the ascertainment of nonhospitalized SVEs. Third, this study only investigated small and null effect sizes; however, unpublished results from an English subset (18 241 of 20 536 [88.8%]) of the Heart Protection Study^46^ found that for major vascular events, routine data follow-up provided very similar effect size estimates (1549 [17.0%] for simvastatin vs 2038 [22.4%] for placebo; RR, 0.74 [95% CI, 0.69-0.79]) compared with adjudicated follow-up (1464 [16.0%] for simvastatin vs 1925 [21.1%] for placebo; RR, 0.73 [95% CI, 0.68-0.78]) (J.A., written communication, September 30, 2021). Fourth, the study required participants to be able to complete mail-based forms, potentially excluding those with impaired cognitive function (which may have increased the reliability of information provided on preadjudicated outcomes). Last, because the ASCEND trial was performed in the UK, which has an NHS, it was unable to assess the reliability of routine data in non-UK countries (including those that do not have a single national health care provider) or whether trials were conducted across different countries. However, the ADAPTABLE team of trialists in the US^44^ have demonstrated the feasibility of a collaborative network (known as the Patient-Centered Clinical Research Network^45^) to perform a large randomized clinical trial of different aspirin doses using electronic health care records as the key method of participant identification and follow-up feasible in a noncentralized health care system.

## Conclusions

The findings of these post hoc analyses of UK routine hospitalization and mortality records linked to the ASCEND trial show how such data can be used to provide reliable estimates of the treatment effect sizes of interventions on myocardial infarctions, hospitalized ischemic strokes, arterial revascularizations, and vascular deaths in primary prevention trials, without the need for verification by clinician adjudicators, if a moderate increase in sample size or follow-up is feasible. However, these routine data sets were not appropriate for capturing TIAs and minor strokes that did not lead to a hospitalization. We therefore recommend for those cerebrovascular outcomes occurring within the community that direct participant follow-up and/or additional data sets such as UK primary care data are used. Using UK routine data for ascertainment of outcomes could substantially streamline within-trial and posttrial follow-up of randomized cardiovascular trials.

## Notes

Supplement 1.
Trial Protocol

Supplement 2. **eTable 1.** Codes Used to Define Any Serious Vascular Event or Revascularization in Routine Data **eTable 2.** Baseline Characteristics of the ASCEND Trial Population **eTable 3.** Agreement of Routine Data vs Adjudicated Direct Follow-up for Any Serious Vascular Event Including Transient Ischemic Attack, by Subgroups **eTable 4.** Agreement of Routine Data vs Adjudicated Direct Follow-up, by Diagnostic Position **eTable 5.** Comparison of Routine Data Event Date vs Adjudicated Direct Follow-up **eTable 6.** Information From Adjudicated Direct Follow-up for Events Only Considered to Be Serious Vascular Events in Routine Data **eTable 7.** Information From Routine Data for Events Only Considered to Be Serious Vascular Events in Adjudicated Direct Follow-up **eTable 8.** Agreement of Routine Data vs Adjudicated Direct Follow-up, Where Adjudicated Data Restricted to Fatal and Hospitalized Event Only **eTable 9.** Comparison of Estimated Treatment Effect Sizes Between Adjudicated Follow-up and Routine Data for the Aspirin and ω-3 Fatty Acids Randomized Comparisons **eFigure 1.** Methods of Follow-up in the ASCEND Trial **eFigure 2.** Association of Allocation to Aspirin vs Matching Placebo and to ω-3 Fatty Acids vs Matching Placebos With Serious Vascular Event or Revascularization Using Routine Data Follow-up, Where Adjudicated Data Restricted to Fatal and Hospitalized Event Only

Supplement 3.
Data Sharing Statement
