# Supplementary material for: Comparison of the Accuracy and Completeness of Records of Serious Vascular Events in Routinely Collected Data vs Clinical Trial–Adjudicated Direct Follow-up Data in the UK: Secondary Analysis of the ASCEND Randomized Clinical Trial
Source: JAMA Netw Open. 2021 Dec 28;4(12):e2139748. doi: 10.1001/jamanetworkopen.2021.39748 (PMC8715347; doi:10.1001/jamanetworkopen.2021.39748)
Supplement: Supplement 1. — Trial Protocol [file jamanetwopen-e2139748-s001.pdf]

ASCEND (A Study of Cardiovascular Events iN Diabetes):
A randomised 2x2 factorial design study of aspirin versus placebo, and of omega-3 fatty acid supplementation versus placebo, for the primary prevention of cardiovascular events in people with diabetes

Should aspirin be used routinely in people with diabetes but no vascular disease?

The role of antiplatelet therapy (chiefly aspirin) for the *secondary* prevention of cardiovascular disease is firmly established for many high-risk groups with diagnosed occlusive arterial disease, and the proportional reductions in heart attacks and strokes appear to be similar whether or not these patients have diabetes. But, most younger and middle-aged people with diabetes do not have manifest arterial disease – although they are still at significant cardiovascular risk – and yet the available randomised evidence for the use of antiplatelet therapy in such individuals is sparse. As a result, there is major uncertainty about the role of antiplatelet therapy for the *primary* prevention of cardiovascular events among people with diabetes, and only a small minority receives it.

ASCEND aims to demonstrate whether aspirin reduces the risk of cardiovascular events in individuals with diabetes who do not already have diagnosed occlusive arterial disease, and whether such benefits outweigh any potential hazards from bleeding. In order to do this reliably, at least 15,000 patients with diabetes and no clinical evidence of occlusive arterial disease will be randomly allocated to receive 100mg aspirin daily or matching placebo tablets for at least 7 years. A study of this size should have excellent power to detect a 12-15% proportional reduction in the cardiovascular event rate among such patients.

Do omega-3 fatty acids (fish oils) reduce cardiovascular risk in people with diabetes?

There is consistent evidence from observational studies of lower rates of cardiovascular disease (particularly cardiac and sudden death) in people with higher intakes, or higher blood levels, of omega-3 fatty acids (FA). Randomised evidence among people who have survived a heart attack suggests modest, but potentially worthwhile, reductions in coronary events of 15-20%. There is, however, no large-scale randomised evidence for the use of omega-3 fatty acids in the primary prevention of vascular events. People with diabetes are at increased cardiovascular risk, and may gain particular benefit from the effects of omega-3 fatty acid supplementation on platelet aggregation and dyslipidaemia. Hence, participants in ASCEND will also be randomly allocated in a 2x2 factorial design to receive 1g omega-3 FA daily or matching placebo capsules for at least 7 years. Such a study design allows all randomised patients to contribute fully to the assessment of the separate effects of aspirin therapy and of omega-3 fatty acids.

ASCEND: A streamlined, mail-based trial collecting only essential data

The reliable assessment of the important questions that ASCEND is addressing requires the randomisation of a very large number of people with diabetes, and their long-term treatment and follow-up. In order to be able to study 15,000 people with diabetes for at least 7 years at low cost, ASCEND is streamlined and being undertaken predominantly by mail (supplemented by central records). If it can reliably demonstrate that aspirin and/or omega-3 fatty acids safely reduces the risk of cardiovascular events and deaths in patients with diabetes who do not have pre-existing occlusive arterial disease, then this would be relevant to some tens of millions of people world-wide (who are currently not receiving such therapy) and could save tens of thousands of lives each year. Consequently the British Heart Foundation is supporting this large streamlined trial.

CONTENTS

Summary of the study aims and design Front cover

1. Background and rationale

Page

1.1 Reliable assessment of the effects of aspirin for primary prevention of cardiovascular events in diabetes mellitus 4

1.1.1 Diabetes mellitus: An increasingly common cause of cardiovascular disease
1.1.2 Lack of reliable evidence for benefit with antiplatelet therapy in patients with diabetes
1.1.3 Aspirin increases the risk of major bleeding (but appears to be relatively safe in diabetes)
1.1.4 Large-scale randomised evidence is required to demonstrate directly that the benefits of aspirin outweigh any risks in people with diabetes
1.1.5 Aspirin 100mg (enteric coated) daily: an effective and well-tolerated antiplatelet regimen

1.2 Reliable assessment of the effects of dietary supplementation with omega-3 fatty acids 7

1.2.1 Higher intake of omega-3 fatty acids is associated with less coronary heart disease
1.2.2 Randomised trials of omega-3 FA supplementation in post MI patients suggests 15-20% reductions in cardiovascular events but there is no information in diabetes
1.2.3 Cardioprotective effects of omega-3 fatty acids may be additional to those of aspirin
1.2.4 Omega-3 fatty acids are considered safe and well tolerated
1.2.5 Need for a large-scale study of omega-3 FA supplementation in people with diabetes

1.3 Mail-based studies for efficiency and cost-effectiveness 9

1.3.1 Previous successful experience of conducting cost-effective randomised trials by mail

2. Plan of investigation

2.1 Study aims: assessments of outcome 9

2.1.1 Primary assessments
2.1.2 Secondary assessments
2.1.3 Tertiary assessments

2.2 Sample size and predicted number of events 10

2.2.1 Random allocation of at least 15,000 patients with diabetes without arterial disease should provide good statistical power to detect plausible effects
2.2.2 Full efficiency of a 2 x 2 factorial design: separate assessment of both study questions without any material effect on non-drug cost or sample size requirements.

2.3 Data and safety monitoring 12

2.3.1 Interim analyses: role of the Data Monitoring Committee and Steering Committee
2.3.2 Monitoring of any serious adverse events believed to be due to the study treatment

2.4 Central coordination 12

2.4.1 Central coordination and local collaboration
2.4.2 Training and monitoring
2.4.3 Supply of study materials
2.4.4 Data handling
2.4.5 Laboratory measurements and sample storage
2.4.6 Source documents and archiving
2.4.7 Source of support and non-negligent liability cover
2.4.8 Publication in the names of all the collaborators

3. SUMMARY OF PRACTICAL PROCEDURES 15

3.1 Eligibility for ASCEND 16

3.2 Identification of participants …………………………………………………………. 16
3.2.1 Large numbers of potentially eligible patients can be identified through diabetes registers, trial databases and general practice

3.3 Screening (- 2 months) ……………………………………………………………………… 17
3.3.1 Establishing eligibility
3.3.2 Pre-randomisation Run-in treatment and optional blood and urine sampling

3.4 Randomisation (0 months) ………………………………………………….…………… 17
3.4.1 Final check of eligibility and compliance before randomisation
3.4.2 Random allocation of aspirin 100mg daily versus placebo, and of 1g daily capsules containing omega-3 fatty acids versus placebo

3.5 Post-randomisation Follow-up …………………………………………………………… 18
3.5.1 6-Monthly follow-up questionnaires sent by mail (with telephone back-up)
3.5.2 Modifying study treatment
3.5.3 Follow-up of deaths and of non-fatal cancers through central registries

3.6 Reporting serious adverse events …………………………………………………….. 18
3.6.1 Immediate reporting of expected and unexpected serious adverse events believed with a reasonable probability to be due to study treatment
3.6.2 Reporting of other serious adverse events on routine follow-up questionnaires
3.6.3 Unblinding of study treatment allocation

3.7 Central ascertainment of biochemical effects and confirmation of reported vascular events, cancers and death ……………………………………………….…….... 19
3.7.1 Assessing biochemical efficacy of study treatments by random sampling
3.7.2 Confirmation of patient reported cardiovascular and other significant serious adverse events using mail-based systems

Appendix 1: ASCEND Information leaflet for potentially eligible patients ……………….. 21
Appendix 2: Consent form ……………………………………………………….………. 25

References 26

Steering and Data Monitoring Committees ……………..………………………. 29

Contact details for Coordinating Centre ………………………….……….……… 29

1. BACKGROUND AND RATIONALE

1.1 Reliable assessment of the effects of aspirin for primary prevention of cardiovascular events in diabetes mellitus

1.1.1 Diabetes mellitus: An increasingly common cause of cardiovascular disease

Diabetes mellitus affects about 150 million individuals worldwide, with at least 40 million cases in the Established Market Economies and over one million diagnosed cases in the UK.[1] Moreover, the prevalence is increasing rapidly, and it is estimated that there will be 300 million people worldwide with type 2 diabetes mellitus by 2025, and a further 30 million with type 1 disease.[2] Patients with diabetes of either type are at substantially increased risk of cardiovascular events and death, and the majority (60-70%) of deaths in both types of diabetes are attributed to vascular causes.[3,4] However, among prevalent cases of diabetes in younger and middle age, the majority will not have a history of vascular disease.[5,6]

1.1.2 Lack of reliable evidence for benefit with antiplatelet therapy in patients with diabetes

In the "secondary" prevention of cardiovascular disease, there is reliable randomised evidence that antiplatelet therapy (chiefly aspirin) reduces the risk of further cardiovascular events by about one-quarter among a wide range of different high-risk groups with occlusive arterial disease,[7,8] and the benefits appear to be similar whether or not such patients also had diabetes (figure 1). As a consequence, most patients with diabetes who have diagnosed vascular disease are currently receiving antiplatelet therapy[9,10] and its use is widely included in guidelines for secondary prevention.[11,12]

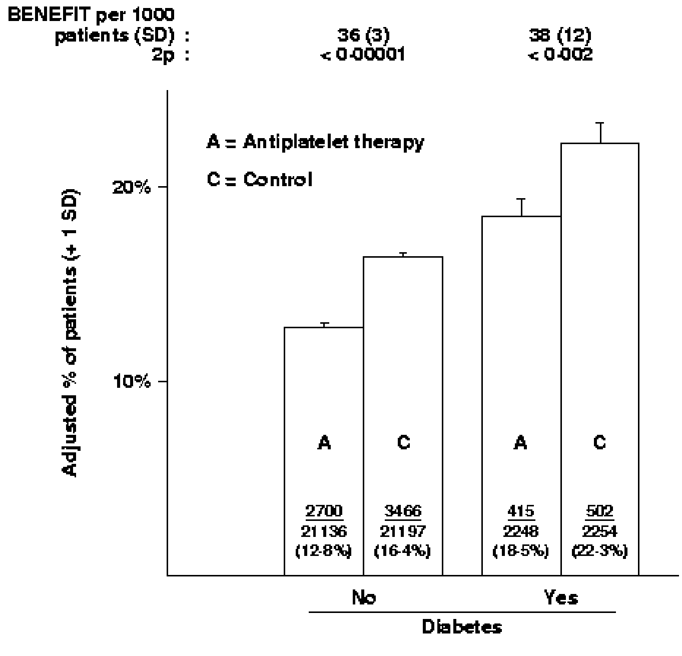

Figure 1: Absolute effects of antiplatelet therapy on vascular events among patients with occlusive arterial disease in the absence and presence of diabetes[7]

However, the majority of people with diabetes do **not** have manifest occlusive arterial disease[5,6] (at least 0.5 million in Britain and several tens of million worldwide), and for them there is no direct evidence of benefit with aspirin or any other antiplatelet agent. The main randomised evidence currently available on the effects of antiplatelet therapy in such patients with diabetes comes from 9 trials involving a total of about 5000 patients, and a meta-analysis of their results

indicates a much smaller proportional reduction in cardiovascular events than has been found in the secondary prevention setting (just 7% compared with about 20-25%: figure 2).[8] Even in aggregate, however, those studies in diabetics involved relatively few events, and the confidence interval for the estimated effect is wide, ranging from a 23% risk reduction to an 8% hazard.

Given the consistency of the beneficial effect in other high-risk settings (including patients with diabetes with arterial disease: figure 1), it seems likely that the true effect of antiplatelet therapy in people with diabetes alone is similar to the reduction of about one-quarter seen overall in high-risk patients as, for example, has been shown with cholesterol-lowering[13] and anti-hypertensive therapies[14]).

Category of patient	No of trials with data	No of patients	Percent with vascular event: Allocated antiplatelet	Percent with vascular event: Adjusted control	%Odds reduction (SE)
Previous myocardial infarction	12	20000	13.5	17.0	25(4)
Acute myocardial infarction	15	19300	10.4	14.2	30(4)
Previous stroke/TIA	21	12000	17.8	21.4	22(4)
Coronary artery disease	55	17500	6.2	8.9	37(5)
High risk of embolism	14	5200	13.5	16.8	26(7)
Peripheral arterial disease	42	9700	5.8	7.1	23(8)
Other vascular conditions	20	3400	4.2	6.4	32(13)
Diabetes mellitus	9	5100	15.7	16.7	7(8)
ALL TRIALS	195	144100	10.7	13.2	22(2)

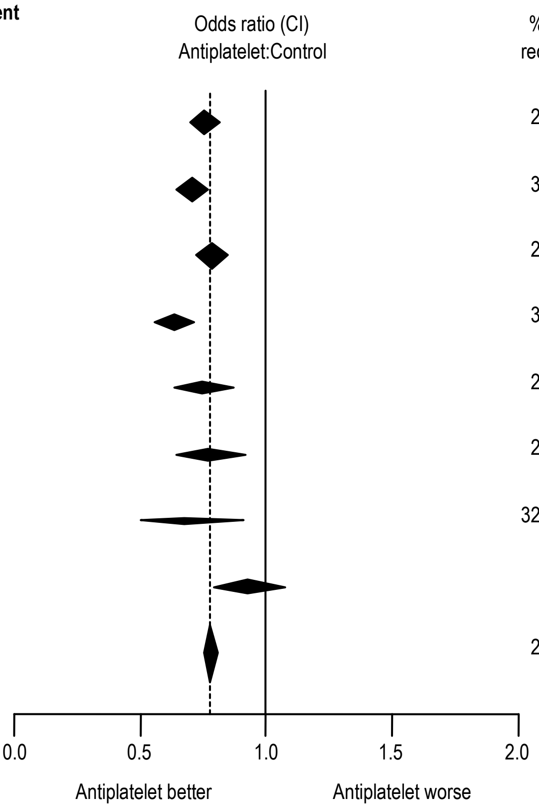

Figure 2: Proportional effects of antiplatelet therapy on vascular events in 195 trials among high-risk patients subdivided by disease category[8]

1.1.3 Aspirin increases the risk of major bleeding (but appears to be relatively safe in diabetes)

In the meta-analysis of previous trials among people with occlusive arterial disease, antiplatelet therapy was found to increase the risk of cerebral haemorrhage by about 25% and the risk of major extracranial bleeds by about 60%, with similar proportional increases in the different types of patient studied.[8] Among such high-risk patients, the absolute reductions in heart attacks and ischaemic strokes with antiplatelet therapy substantially outweighed the relatively small absolute risks of cerebral haemorrhage and major extracranial bleeds. There is also good evidence from the previous trials that antiplatelet therapy is not associated with any special risks in patients with diabetes. In particular, the Early Treatment Diabetic Retinopathy Study (ETDRS) of 650 mg aspirin daily versus placebo among 3700 people with diabetes indicated that aspirin did not increase the risk of retinal or vitreous haemorrhage.[15] Nevertheless, there is a lack of reliable direct evidence that the balance of benefits and risks of antiplatelet therapy among patients with diabetes alone is favourable.

1.1.4 Large-scale randomised evidence is required to demonstrate directly that the benefits of aspirin outweigh any risks in people with diabetes

The emergence of reliable evidence about the substantial net benefits produced by aspirin in people with occlusive arterial disease has rapidly lead to its widespread use in such patients (with, for example, over 80% of those with a history of previous heart attacks or strokes receiving some form of antiplatelet therapy).[10] Based on extrapolation from the evidence in these other high-risk settings, the American Diabetes Association (ADA) has recommended the use of aspirin in people with type 2 diabetes and at least one additional risk factor (e.g. hypertension or hypercholesterolaemia).[16] By contrast, UK and European guidelines are more circumspect in their recommendations about aspirin use for people with diabetes alone.[11,12] Presumably as a result of the current uncertainties about the net benefit of antiplatelet therapy in this setting, surveys in the US and UK indicate that only about 10-20% of patients with diabetes without diagnosed occlusive arterial disease are taking antiplatelet therapy regularly.[17,18] Similarly, less than 20% of diabetic patients without vascular disease were taking aspirin regularly in the United Kingdom Prospective Diabetes Study (UKPDS) and the MRC/BHF Heart Protection Study[19] (HPS) conducted in Britain, as well as in the ongoing FIELD trial conducted in Australia, New Zealand and continental Europe. Data from the Anglo-Scandinavian Cardiac Outcomes Trial (ASCOT) also indicate that less than 20% of the hypertensive patients with diabetes and no occlusive vascular disease were taking aspirin in the last 6 months of the study in Sweden, Denmark and Norway (personal communication).

Currently, the only ongoing comparison of antiplatelet therapy versus no antiplatelet therapy in patients with diabetes without pre-existing occlusive arterial disease involves 2000 of the participants in the Women's Health Study (WHS), which is too few to assess the effects of treatment in such individuals reliably (see below). The Prevention of Progression of Asymptomatic Diabetic Arterial Disease (POPADAD)[20] study involves the assessment of aspirin among a further 1600 patients with diabetes, but all of the participants in that trial have diagnosed peripheral arterial disease. Further information about the effects of antiplatelet therapy among diabetic patients without pre-existing arterial disease will emerge from a collaborative meta-analysis of individual participant data from all of the previous "primary" prevention aspirin trials. But, preliminary results among the 3000 low-risk diabetic participants involved in that analysis indicate only a non-significant 25% (SD 16) reduction in coronary events (59 [3.9%] aspirin-allocated versus 71 [4.9%] placebo-allocated events; 2P=0.1) during median follow-up of 5 years (personal communication). Hence, there is a real need to initiate a much larger randomised trial of antiplatelet therapy in people with diabetes without occlusive arterial disease for whom there is not considered to be any clear indication for such treatment.

1.1.5 Aspirin 100mg (enteric coated) daily: an effective and well-tolerated antiplatelet regimen

The Anti-Thrombotic Trialists' (ATT) collaborative meta-analysis of previous trials found that high doses of 500-1500mg aspirin daily (which are more gastrotoxic[21]) are no more effective than lower doses of 75-100mg/day either in direct comparisons or in indirect comparisons (Figure 3).[7] As a consequence, daily doses of 75-150mg are generally preferred for long-term treatment as protection against serious vascular events in high-risk patients. The use of enteric-coating delays the dissolution of the contents of the tablet until the higher pH of the duodenum is reached, and so may reduce gastric injury and symptoms.[22] Hence, a regimen of 100mg daily enteric-coated aspirin is to be used in this study.

Aspirin dose mg/day	No of trials with data	No of patients	Percent with vascular event: Allocated aspirin	Percent with vascular event: Adjusted control	%Odds reduction (SE)
500-1500	34	22500	14.5	17.2	19(3)
160-325	19	26500	11.5	14.8	26(3)
75-150	12	6800	10.9	15.2	32(6)
<75	3	3700	17.3	19.4	13(8)
Any aspirin	65	59400	12.9	16.0	23(2)

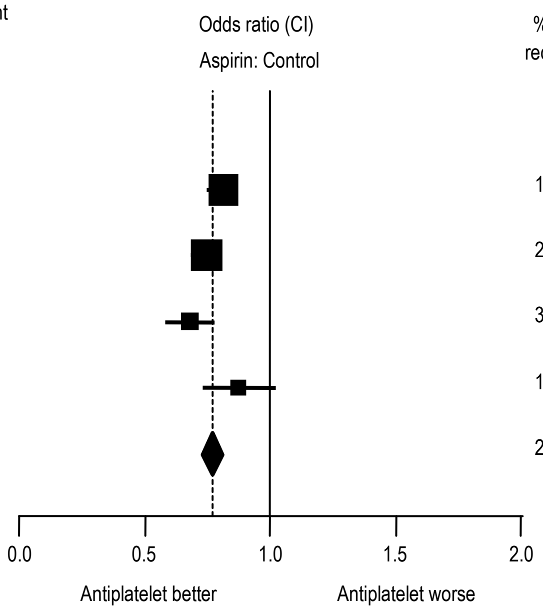

Figure 3: Proportional effects of different doses of aspirin on vascular events in high-risk patients[7]

Protocol addition December 2017

Aspirin and cancer

In the years since ASCEND was designed it has become apparent that aspirin therapy may also reduce the incidence of cancer. If this benefit is confirmed in prospective randomised trials, it has important implications for establishing the balance of benefits and risks of aspirin therapy.

Based on a series of post-hoc analyses of the long-term follow-up of certain randomised trials of aspirin, and of observational studies of cancer incidence in relation to aspirin use, it has been suggested that aspirin protects against various forms of cancer, but that this effect takes some years to become apparent. (Flossmann et al Lancet. 2007; 369: 1603-13, Rothwell et al. Lancet. 2010; 376: 1741-50; Lancet. 2011; 377: 31-41; Lancet. 2012; 379: 1602-12 and Lancet. 2012; 379: 1591-601; Algra et al Lancet Onc. 2012; 13: 518-27 Jacobs et al. J Nat Can I. 2012; 104: 1208-17 Downer et al Eur Urol. 2017 and Cook et al Ann Int Med. 2013; 159: 77-85). In one early analysis of data from 2 randomised trials, aspirin allocation scheduled for 5 or more years reduced the 20-year risk of colorectal cancer with a hazard ratio [HR] of 0.63 (95% CI 0.47-0.85), with no effect of aspirin seen for the first 10 years of follow-up. A later analysis by the same authors including 6 additional trials, indicated that aspirin allocation scheduled for 5 or more years was associated with a 20% lower risk of death from any cancer after 20 years; HR 0.78 (0.70-0.87). This effect was driven by reductions in colorectal and oesophageal cancer deaths leading to a 35% reduction in any gastrointestinal cancer deaths (HR 0.65 [0.53-0.78] based on 400 events) and a reduction in lung cancer deaths. The reduction was seen during the first 10 years, as well as between 10 and 20 years of follow up. The lag period before an effect on deaths was observed was around 5 years for oesophageal, pancreatic, brain, and lung cancer, but was longer for stomach, colorectal, and prostate cancer. In 2012, the same authors in further analyses of largely the same trials argued that aspirin reduced the risk of cancer incidence even during the scheduled treatment period of the trials; HR 0·88 (0·80–0·98), with no effect during the first 3 years of follow-up, but benefit increasing with duration of follow-up; 0–2·9 years, HR 1·00 (0·88–1·15); 3–4·9 years, HR 0·81 (0·67–0·98); ≥5 years HR 0·71 (0·57–0·89). Similar results for colorectal cancer have been observed in one large US trial of alternate day aspirin after 20 years; HR 0.80 (0.67–0.97) but with no impact on overall cancer incidence HR 0.98 (0.90-1.07).

The ASCEND trial provides one of the first opportunities to prospectively test the hypothesis that aspirin prevents gastrointestinal and overall cancer incidence and death, both during the trial and during planned longer term post-trial follow-up (see below). For the in-trial analysis the primary cancer endpoint will be any gastrointestinal (GI) tract cancer. Little or no treatment effect is expected before about 3 years (based on the earlier trial data) therefore limiting the statistical power to detect plausible effects of aspirin during the scheduled treatment period. The expected ~430 GI tract cancers during this period provide ~86% power at 2p<0.05 to detect a 40% reduction in risk and 60% power at 2p<0.05 for a 30% reduction in risk. Analyses by time from randomisation will help assess whether effects are increasing with duration of aspirin use.

However, the main focus of the cancer analyses will be during planned longer term follow-up, when there will be much better power to detect plausible differences between the arms due to larger numbers of events. At about 5 years after the scheduled treatment period, there will be >90% at 2p<0.01 to detect a 30% or greater risk reduction and >90% at 2p<0.05 to detect a 25% reduction in any GI cancer risk (see Data Analysis Plan).

Long-term follow-up

Ethics approval was obtained during 2017 to continue to collect health-related information from ASCEND participants beyond the end of the scheduled treatment period for at least a further 20 years. Information about deaths, cancers and hospitalisation for any reason will continue to be collected from centrally held electronic records via NHS digital (formerly the Health and Social Care Information Centre). Planned analyses are described in the Data Analysis Plan.

1.2 Reliable assessment of the effects of dietary supplementation with omega-3 fatty acids

1.2.1 Higher intake of omega-3 fatty acids is associated with less coronary heart disease

Omega-3 fatty acids are long-chained polyunsaturated fatty acids (PUFA) with their first double-bond found at the third carbon atom from the methyl group (which is why they are referred to as n-3 or omega-3 fatty acids). Man is unable to manufacture these omega-3 fatty acids (FA) and is reliant upon intake from plants and animals. The richest dietary sources of the two principal omega-3 fatty acids, eicosapentaenoic acid (EPA; 20:5n-3) and docosahexaenoic acid (DHA; 22:6n-3), are marine animals.[23] Consumption of oily fish 2-3 times per week provides about 500mg daily of EPA and DHA combined, but consumption is less than about 50mg per day in people who do not eat fish regularly.[24]

The possible link between high intake of omega-3 FA and prevention of coronary heart disease was first noted in the 1940s when the diets of Greenland Eskimos, among whom coronary disease was rare, were compared with those of Danes living in Denmark where coronary heart disease (CHD) rates were about 10 times higher.[25] Despite similar total fat intake (about 40% of total calories), eskimo diets contained significantly greater proportions of omega-3 FA (>4%) compared with the Danes (<0.1%). These observations stimulated a large number of observational studies of omega-3 FA intake and heart disease risk in different populations. A 1999 systematic review of all of the observational data concluded that in high-risk populations consumption of the equivalent of 40-60 grams of fish per day (providing about 0.2-1g daily of omega-3 FA depending on the type of fish) is associated with 40-60% lower rate of cardiac death.[26] More recently, other observational studies have found similar protective associations of fish consumption and incidence of CHD,[27-29] (including among 5000 women with diabetes followed for about 9 years[30]) and stroke.[24]

1.2.2 Randomised trials of omega-3 FA supplementation in post-MI patients suggest 15-20% reductions in cardiovascular events but there is no information in diabetes

In the only large randomised trial of omega-3 FA supplementation that has been conducted to date, 11,000 heart attack survivors in Italy were allocated to receive 1g daily of n-3 PUFA (containing 0.46g of EPA and 0.38g of DHA) versus no PUFA treatment for 3.5 years.[31] Marginally significant reductions of 13% (95% CI 1-24%, p=0.04) in coronary events (i.e. non-fatal myocardial infarction [MI] or coronary death) and of 17% (95% CI 3-29%, p=0.02) in cardiovascular deaths, were observed among those allocated PUFA capsules in this GISSI-Prevenzione trial. This was despite 80-90% of patients in both groups eating fish at least once a week, and high use of cardioprotective drugs (including aspirin). In another randomised trial, 2000 men with a history of myocardial infarction in Wales were allocated to a recommended intake of at least 2 portions of fatty fish per week (or 1.5g Maxepa capsules daily, which contain about 0.5g EPA) versus no change in fish intake for 2 years.[32] There was a non-significant trend towards 17% fewer (95% CI 35% reduction to 8% excess) coronary events among patients allocated increased fish intake, and cardiac deaths were by 35% (95% CI 13-52%, p=0.004). Background intake of fish in that Welsh population was low, and only about 10% of the patients were taking aspirin. In a meta-analysis of all of the available unconfounded randomised evidence for increased omega-3 FA intake from these two trials and 9 much smaller trials[33,34] (which tested doses of EPA and DHA in the range 1-6g per day among a total of about 2000 patients), there was a highly significant reduction in coronary events of 18% (95% CI 8-27%, p=0.0008). Based on these studies – which were conducted chiefly among people with vascular disease – it would seem plausible that omega-3 FA supplementation might produce a 15-20% reduction in coronary and other occlusive vascular events among high or intermediate risk populations, including people with diabetes.

1.2.3 Cardioprotective effects of omega-3 fatty acids may be additional to those of aspirin

Aspirin irreversibly inhibits platelet cyclo-oxygenase, the enzyme that controls the conversion of arachidonic acid to prostaglandins and thromboxanes, which reduces the formation of thromboxane A2 in platelets and produces a potent anti-aggregatory effect.[21] But, aspirin also reduces the formation of prostacyclin, which is a potent vasodilator, and so may lead to vasoconstriction. Omega-3 FA (particularly EPA) compete with arachidonic acid for cyclo-oxygenase,[23] and so reduce thromboxane A2 production in platelets (albeit to a lesser extent than aspirin). Unlike aspirin, however, omega-3 FA enhance prostacyclin production in endothelial cells. Moreover, when aspirin and omega-3 FA are given together, there is a shift towards increased prostacyclin formation in endothelial cells and vasodilatation.[35] Consequently, any beneficial effects of aspirin and omega-3 FA on vascular disease that are mediated through these effects on prostaglandins and thromboxanes should be complementary.[35] Omega-3 FA might also have other cardioprotective effects, including: reducing myocardial susceptibility to ventricular arrhythmias;[36] increasing the stability of atherosclerotic plaques through anti-inflammatory effects that are mediated by prostaglandins and leukotrienes;[37] reducing blood pressure;[38] and reducing plasma concentrations of triglycerides (TG) and very-low-density lipoproteins, and inhibiting post-prandial lipaemia.[39-41] These effects of omega-3 FA on lipoproteins are seen both in the presence, and in the absence, of statin therapy.[34] As cardiovascular disease in diabetes derives both from platelet activation[42] and from disordered triglyceride metabolism,[3] omega-3 FA may be particularly worthwhile for people with diabetes.

1.2.4 Omega-3 fatty acids are considered safe and well tolerated

The Food and Drug Administration (FDA) consider omega-3 FA doses of up to at least 3g daily to be safe,[23] with no significant risk of bleeding. In the large GISSI Prevenzione trial,[31] 90% of participants were taking aspirin, but no excess of bleeding was observed with the addition of 1g

omega-3 FA daily. The only side-effects reported in that open-label study were a slight fishy after-taste and some gastrointestinal disturbances, but only 3.8% of participants stopped their omega-3 FA supplements because of these side effects. Omega-3 FA have no effect on glycaemic control in diabetes[40,43] and their small, potentially adverse, effects on plasma concentrations of LDL-cholesterol may be offset by beneficial changes in lipoprotein particle size.[39,41] For the present trial, a daily dose of approximately 1g capsules containing omega-3 FA (0.46g EPA and 0.38g DHA is to be used (as in GISSI)), which can be conveniently provided in 1 capsule of the concentrated preparation (with matching placebo capsules containing olive oil).

1.2.5 Need for a large-scale study of omega-3 FA supplementation in people with diabetes

As discussed above, diabetes is associated with a 2-4 fold increase in the risk of cardiovascular disease and the incidence of diabetes worldwide is increasing rapidly. Consequently, the demonstration that an inexpensive and readily available food supplement – such as omega-3 FA – reduces cardiovascular risk in patients with diabetes would have important public health consequences. By adopting a 2x2 factorial design within this large streamlined study, it will be possible to assess the separate and combined effects of both aspirin and omega-3 FA supplementation in a particularly cost-effective manner.

1.3 Mail-based studies for efficiency and cost-effectiveness

1.3.1 Previous successful experience of conducting cost-effective randomised trials by mail

Both aspirin and omega-3 FA are widely available and used, the hazards are low and well characterised, and neither requires biochemical monitoring. Several large randomised trials have been conducted using mailed drug supply and follow-up, including the CTSU-coordinated British Doctors' Study[44] and the (first) US Physicians Health Study[45] of aspirin for the prevention of myocardial infarction. Currently, there are 3 large studies[46-48] of either aspirin or various supplements being conducted entirely by mail in the US: the (second) US Physicians' Health Study II, the Women's Antioxidant Study (WACS) and the Women's Health Study (WHS). The latter study includes 40,000 American women from a wide range of educational and social backgrounds randomised to aspirin or matching placebo, and in a factorial design to different vitamin and mineral combinations. Experience from these studies shows that - with appropriate attention to the wording of information leaflets, consent forms and questionnaires, - good response rates and compliance can be achieved and reliable information about medical events gathered.[49] In addition, the 24-hour Freefone service established by CTSU for other large heart disease trials will allow study participants to discuss any aspects of the study with experienced clinical staff, and so help ensure good compliance and the early identification of serious problems.

2. PLAN OF INVESTIGATION

2.1 Study aims: assessment of outcomes

The aim of ASCEND is to determine whether 100mg daily aspirin and/or supplementation with 1 gram capsules containing 90% omega-3 fatty acids (0.46g EPA, 0.38g DHA) daily prevents cardiovascular events in patients with diabetes who do not already have clinically manifest arterial disease (without leading to significant bleeding or other adverse events).

2.1.1 Primary assessments

Aspirin therapy: The primary efficacy comparison will involve "logrank" analyses[54] of "serious vascular events" (defined as the combination of non-fatal myocardial infarction, non-fatal stroke or transient ischaemic attack, or vascular death excluding confirmed cerebral haemorrhage during the scheduled treatment period among all those allocated aspirin tablets versus all those allocated placebo tablets (i.e. "intention-to-treat" comparisons). (Vascular death includes ICD I00-I52 and I63-99 in the 10th International Classification of Diseases.)

The primary safety assessment will involve intention-to-treat comparisons among all randomised patients of allocation to aspirin versus placebo on the first occurrence of "any major bleed", defined as: the incidence of any "major haemorrhage" (defined as any major intracranial haemorrhage, sight-threatening eye bleeding or any other bleeding episode that requires hospitalisation or transfusion, or is fatal or disabling)

Omega-3 fatty acid supplementation: The primary comparison will involve "logrank" analyses of "serious vascular events" during the scheduled treatment period among all those allocated omega-3 fatty acid capsules versus all those allocated placebo capsules.

2.1.2 Secondary assessments

The principal subsidiary comparisons will be of the effect of allocation to aspirin versus allocation to placebo tablets and, separately, of allocation to omega-3 FA versus allocation to placebo capsules on: the incidence of the combined endpoint of "serious vascular events (SVE) or revascularisations" (i.e. serious vascular event, or coronary or non-coronary revascularisation)

For the aspirin comparison only, secondary efficacy and safety assessments include:
Any incident gastrointestinal (GI) tract cancer (i.e any GI cancer excluding pancreas and hepatobiliary); and the first occurrence of:

i. haemorrhagic stroke (i.e. intracerebral or subarachnoid haemorrhage), overall and by level of disability (fatal; disabling; non-disabling; unknown disability);

ii. any major bleed by site:
- intracranial haemorrhage and separately its components (intracerebral, sub-arachnoid, subdural and other haemorrhage);
- sight-threatening eye bleed;
- serious gastrointestinal (GI) haemorrhage;
- other serious bleed (ie any extra-cranial, extra-ocular or non GI haemorrhage).

2.1.3 Tertiary and exploratory assessments

In addition, comparisons will be made of the effects during the scheduled treatment period of each of the study treatment allocations on: total and cause-specific mortality (coronary, other vascular and non-vascular death separately); other vascular outcomes (eg any coronary events (ie. non-fatal myocardial infarction, coronary death or coronary revascularisations [i.e. CABG and PTCA]); non-haemorrhagic strokes or transient ischaemic attacks); microvascular complications; venous thromboembolism; total and site-specific cancers; and hospitalisations for various other causes as indicated in the Data Analysis Plan. In addition, while it is not anticipated that the proportional effects of aspirin or omega-3 FA on particular outcomes will vary depending on particular baseline characteristics these will be explored in various prognostic subgroups (see Data Analysis Plan). Allowance for multiple hypothesis testing will be made in the interpretation of these analyses.

2.2 Sample size and predicted number of events

2.2.1 Random allocation of at least 15,000 patients with diabetes without arterial disease should provide good statistical power to detect plausible effects

One particular cohort of people with diabetes and no evident cardiovascular disease had a coronary event rate of around 3% per annum.[55] However, although that study is widely quoted, event rates may not be as high in diabetic populations with lower levels of other risk factors. For example, among the 5000 men and women with newly diagnosed type 2 diabetes (mean age 53 years) randomised in the UKPDS,[56] annual rates were 1.6% for coronary events and 1.1% for death due to macrovascular disease (i.e. fatal MI, stroke or sudden death). Similarly, among about 3000 diabetics (mean age 63) without diagnosed occlusive arterial disease randomised in HPS,[13,57] the annual overall rate of cardiovascular events (fatal or non-fatal myocardial infarction or stroke) was 2.2%. Hence, it would seem prudent to base sample size calculations for any randomised trials in patients with diabetes and no arterial disease on serious vascular event rates of no more than about 2% per annum.

Aspirin has been shown to reduce cardiovascular event rates by about one quarter in a wide range of high-risk groups with arterial disease, with similar proportional reductions irrespective of whether or not diabetes is present (see Figure 1).[7] Hence, as in other high-risk populations, it seems plausible that aspirin might reduce the risk of serious vascular events by around one-quarter in patients with diabetes who do not have clinical evidence of arterial disease. Similarly omega-3 FA have reduced risk of cardiovascular events by 15-20% in high-risk populations. Proportional reductions of 15-20% among diabetics without diagnosed arterial disease would still correspond to substantial absolute benefits (see Table 1). But, even if such benefits do exist, at least 10,000 patients with diabetes would need to be randomised and followed for 5 years to detect these effects reliably. During the trial it is intended that blinded event rates (i.e. active and placebo groups combined) will be monitored and, if they are substantially lower than anticipated, the Steering Committee will have the option of increasing the sample size or prolonging the scheduled treatment period (see below)

Table 1: Statistical power to detect 15-20% proportional reductions in serious vascular events among 10,000 randomised patients (based on 10% 5 year control group event rate)

Proportional reduction	Control group 5000	Active group 5000	Power at 2P<0.01	Power at 2P<0.05	Events avoided/ 1000 over 5 years
25%	500	375	>95%	>95%	25
20%	500	400	80%	>90%	20
15%	500	425	60%	70%	15

Protocol addition January 2011: Accumulating evidence from within ASCEND, suggests that the overall (i.e. blinded) annual event rate (including transient ischaemic attacks) is likely to be about 1.2%, i.e. somewhat lower than the initial estimate of 2% pa in the control group. In addition, a recent meta-analysis of primary prevention trials of aspirin suggests that reductions in serious vascular events of 12-15% may be more likely than reductions of 20-25%.[58] With an annual event rate of 1.2% in the control group, randomisation of 15,000 patients with follow-up of 7.5 years would provide robust statistical power to detect plausible risk reductions of 12-15%. Hence, the Steering Committee agreed during 2010 to increase the sample size to 15,000 and the duration of follow-up to at least 7 years.

Protocol addition December 2017: A total of 15480 patients were randomised between 2005 and 2011 and have been followed for 7.5 years. Based on the blinded overall event rate for the primary outcome, the study has ~90% power at $p<0.05$ to detect a proportional reduction of 15% in risk of the primary endpoint.

2.2.2 Full efficiency of a 2 x 2 factorial design: separate assessment of both study questions without any material effect on non-drug cost or sample size requirements.

A factorial design will be used, with at least 7500 patients being randomly allocated to receive aspirin tablets versus 7500 patients allocated to receive matching placebo tablets (see figure 4). Similarly, at least 7500 patients will be separately randomised to receive omega-3 FA capsules versus 7500 patients allocated to receive placebo capsules. The primary analyses will involve two-way comparisons of all those allocated aspirin versus all those allocated matching placebo tablets, irrespective of the omega-3 FA allocation (Figure 4: subtotal A versus subtotal B), and of all those allocated omega-3 FA versus all those allocated matching placebo capsules irrespective of the aspirin allocation (i.e. subtotal 1 versus subtotal 2). Hence, reliable assessment of the effects of aspirin will not interfere with reliable assessment of omega-3 FA (or vice versa), as outcomes among all those allocated active aspirin can still be compared with those among all those allocated placebo aspirin (even though half of each group will have received omega-3 FA). Use of such a factorial design instead of a simple 2-way design has little or no effect on the statistical sensitivity with which the overall effects can be assessed, or on the total number of patients required in the study.[53]

Figure 4: Factorial design of trial

	**Aspirin Tablets**	**Placebo Tablets**	
**Omega-3 FA capsules**	3750 Aspirin + Omega-3 FA	3750 Omega-3 FA	Subtotal 1: 7500 **Omega-3 FA**
**Placebo capsules**	3750 Aspirin	3750 Neither	Subtotal 2: 7500 **Placebo**
	Subtotal A: 7500 **Aspirin**	Subtotal B: 7500 **Placebo**	

2.3 Data and safety monitoring

2.3.1 Interim analyses: role of the Data Monitoring Committee and Steering Committee

During the study, the independent Data Monitoring Committee will review unblinded interim analyses, at least annually, of mortality, of cardiovascular events and of other serious adverse events, along with any other analyses requested. In the light of these analyses and the results of any other relevant trials or meta-analyses of trials, the Data Monitoring Committee will advise the Steering Committee if, in their view, the randomised comparisons in the study have provided **both** (a) "proof beyond reasonable doubt"* that for all patients, or for some specific

* Appropriate criteria of proof beyond reasonable doubt cannot be specified precisely, but in general a difference of **at least** 3 standard deviations in an interim analysis of a major endpoint would be needed to justify halting, or modifying, such a

types, aspirin therapy is clearly indicated or clearly contraindicated in terms of a net difference in mortality, **and** (b) evidence that might reasonably be expected to influence materially the patient management of many clinicians who are already aware of any other main trial results. The Steering Committee can then decide whether to end or modify the study (or to seek extra data). Unless this happens, the Steering Committee, the collaborators and the coordinating centre staff (except those who supply the confidential analyses) will remain ignorant of the interim results on mortality and morbidity until the study is terminated. Collaborators, and all others associated with the study, may write (preferably through the Oxford coordinating centre) to the chairman of the Data Monitoring Committee, drawing attention to any worries they may have about the possibility of particular side-effects, or about particular categories of patient requiring special consideration, or about any other matters that may be relevant.

2.3.2 Monitoring of any serious adverse events believed to be due to the study treatment

Throughout the trial, all serious adverse events believed with a reasonable probability to be due to study treatment are to be reported immediately by telephoning the 24-hour telephone service (see Section 3.6). A "serious" adverse event is defined as any untoward medical occurrence which results in death, is life-threatening, requires hospitalisation or the prolongation of existing hospitalisation, results in persistent or significant disability or incapacity, or is a congenital anomaly or birth defect. During this telephone call, standard information (i.e. identity of the patient and of the person reporting the event, nature and date of event, and reasons for attribution to study treatment) will be recorded directly on the coordinating centre computer. These reports will be reviewed immediately, blind to treatment allocation, by the clinical coordinators, and any further information required sought urgently. Confirmed reports will then be promptly forwarded "unblinded" to the chairman of the Data Monitoring Committee. and to Bayer Healthcare AG or to Abbott Products Operations AG, as appropriate and included in the Annual Safety Report sent to the Research Ethics Committee (REC). Any such serious adverse events that are also unexpected will be reported in an expedited fashion to the REC and relevant drug regulatory agencies.

2.4 Central Coordination

2.4.1 Central coordination and local collaboration

The Clinical Trial Service Unit (CTSU) at Oxford University is coordinating this study and will have overall responsibility for the administration and coordination of the study. There will be a Steering Committee to oversee the trial conduct (back page). CTSU is responsible for obtaining Multicentre Research Ethics Committee approval; for the training and monitoring of all staff directly involved in the study; for the supply of conveniently packaged study drugs and other study materials; for the identification, with the assistance of the local medical collaborators, of potentially eligible participants; for obtaining any relevant permissions to invite suitable patients to participate; for the initial invitation of participants and subsequent randomisation and follow-up by mail; for the provision of a 24-hour Freefone telephone service (for queries from participants or medical staff, for unblinding when medically necessary, and reporting of any serious adverse events believed to be due to study treatment); and for the collection and analysis of data, and blood samples. The medical collaborators around the UK are responsible, with the help of the Oxford coordinating centre, for obtaining local ethics committee approval and for assisting in the identification of potentially eligible individuals with diabetes (including liaison with hospital medical records staff). (This is summarised below, and is described in detail in the coordinating centre standard operating procedures [SOP].)

---

study prematurely, especially if the comparison was based on relatively few events (e.g. less than 100). If this criterion were to be adopted, it would have the practical advantage that the exact number of interim analyses would be of little importance, and so no fixed schedule is proposed [53].

2.4.2 Training and monitoring

The administrative and nursing staff in the Oxford coordinating centre will be trained in correct study procedures (as summarised in Section 3 of the protocol and described in detail in the SOPs). The coordinating centre staff will also arrange regular meetings of all the collaborators to discuss the progress of the study and other general issues, and to provide an update on the results of any other relevant studies. Collaborators will be encouraged to contact the coordinating centre office (or 24-hour telephone service for urgent queries) if they wish to discuss some problem or other issue related to the study.

2.4.3 Supply of study materials

Aspirin and matching placebo tablets are to be manufactured and provided by Bayer Healthcare AG. Omega-3 FA capsules and matching placebo capsules are to be provided by Abbott Products Operations AG (formerly Solvay Pharmaceuticals). Both treatments are to be delivered in bulk to Brecon Pharmaceuticals for packaging and labeling prior to dispatch to participants. All study medication will be supplied in convenient treatment packs appropriate for mailing which contain the appropriate number of blister-strips for each period of the study. An inventory of study drug supply will be maintained on the coordinating centre computer, and any study drug not required by participants is to be returned to the coordinating centre for disposal.

2.4.4 Data handling

Lists of potentially eligible people with diabetes will be sought, preferably in computerized format, by the Oxford coordinating centre from medical collaborators who have access to diabetes registers, from trial databases and from general practitioners (GPs). This information will be used by the coordinating centre to generate invitations, in the name of the local medical collaborator, for patients to join the study (see Section 3.3). Hospital collaborators, general practitioners or practice nurses will also be able to offer a standard “invitation pack” (containing patient information leaflet, screening questionnaire and freepost envelope) to potentially eligible participants when they are seen for routine care in their clinic. In addition, randomised participants will have the option to recommend any friend or relative they think may be eligible and interested in participating in the study and potential participants can volunteer themselves if they hear about the study from any source. Responses from participants will be collected on questionnaires which are to be returned to the coordinating centre either on paper or electronically. Any coding that is required will be undertaken and data will then be entered into the coordinating centre computer (following an operating procedure for data handling). Any failure by participants to return Follow-up questionnaires will result in two mailed reminders being sent. Subsequently, when necessary, study coordinating staff will undertake a telephone follow-up.

Errors or omissions in the completion of study forms will result, if appropriate, in computer-generated correction requests being sent to participants for completion. All such corrections to the data will be entered on the central computer with an appropriate audit trail. The coordinating centre is also responsible for seeking confirmation and additional information about any relevant clinical events reported during follow-up, and for obtaining details from national registries of any deaths, non-fatal cancers or other relevant events available among study participants (see Section 3.7).

2.4.5 Laboratory measurements and sample storage

Blood and urine samples taken at GP practices from those patients who agree to start Run-in treatment (Section 3.3.2) and samples taken from a randomly selected group of patients during follow up (Section 3.3.1) will be mailed to the coordinating centre laboratory in Oxford. The central laboratory will use part of each blood sample for immediate assays, with the remainder being frozen for subsequent assays. The laboratory uses a number of internal and external

quality control procedures and follows a standard operating procedure (in accordance with Good Laboratory Practice guidelines). Checked assay results will be transferred to the central computer and linked to the patients' other data.

2.4.6 Source documents and archiving

The lists of potentially eligible patients provided to the Oxford coordinating centre by collaborators, the returned questionnaires from these patients, the additional information obtained on reported outcome measures and other relevant events, the death certificates, the blood assay results and the drug supply records constitute the "source documents" for the study. The coordinating centre will retain these data and records for at least 15 years. Regulatory agencies and the companies providing the study medications will have the right, in accordance with Good Clinical Practice guidelines, to commission a confidential audit of such records kept in the coordinating centre, as long as this does not result in unblinding of the interim results while the study is still in progress.

2.4.7 Source of support and non-negligent liability cover

Funding has been obtained from the British Heart Foundation to cover the administrative and coordination costs of the trial. A supply of aspirin and matching placebo is to be provided by Bayer Healthcare AG, and a supply of omega-3 FA and matching placebo capsules by Abbott Products Operations AG (formerly Solvay Pharmaceuticals), with some funding from each company to cover drug packaging.

The trial is to be conducted, analysed and interpreted by CTSU entirely independently of the funding sources, which have no representation in its organisation and will, like the Steering Committee, remain blind to the main results as they accumulate. This arrangement is intended to ensure that no suggestions of lack of objectivity of the findings can be justified.

2.4.8 Publication in the names of all the collaborators

The success of this study depends on the wholehearted collaboration of a large number of doctors, nurses and patients. For this reason, chief credit for the main results will be given not to the central organizers, but to all those who have collaborated in the study. Draft copies of any manuscripts will be provided to all collaborators for review prior to their publication and will be published in the name of the collaboration.

3. SUMMARY OF PRACTICAL PROCEDURES

POTENTIALLY ELIGIBLE

- Diabetes mellitus (type 1 or 2)
- Male or female
- No diagnosed occlusive arterial disease
- Aged ≥ 40 years

IDENTIFICATION & INVITATION

- Potentially eligible patients identified from existing diabetes registers or databases and other sources
- Invited by GP, diabetologist or study coordinators, either in person or by mail. Invitation includes Information Leaflet, Consent Form and brief Screening Questionnaire
- Central Freefone number for any questions

SCREENING PROCESS (-2 months)

- Screening Questionnaire returned, which identifies eligible and consenting patients
- Run-in pack with 2-month supply of placebo treatment mailed to patient
- GP informed of patient's possible participation, and asked to return form if patient **not** to be randomised
- Blood and urine samples (optional) collected locally and mailed to central laboratory
- Freefone number (0800 585323) for medical advice and any questions

RANDOMISATION (0 months)

- Randomisation Questionnaire sent to re-confirm eligibility, and to characterize the patient more fully
- Randomisation Questionnaire returned, and eligible patient randomised by central computer
- Allocated treatment pack mailed to patient: 100 mg aspirin daily or matching placebo tablet, and 1g capsule containing omega-3 FA daily or matching placebo
- GP informed of patient's randomisation

FOLLOW-UP QUESTIONNAIRES (6-monthly)

- Follow-up Questionnaires and treatment packs sent 6-monthly
- Freefone number (0800 585323) for medical advice and any questions
- Further details sought from responsible clinicians about any relevant events reported on Follow-up questionnaires
- Flagging for mortality and cancer at central registries

3.1 Eligibility for ASCEND

Men or women aged at least 40 years at the time of invitation for Screening are eligible for the study, provided they fulfil **all** of the following criteria:

- **Clinical diagnosis of diabetes mellitus:** The participant's own doctor considers them to have type 1 or type 2 diabetes (based on standard WHO or ADA diagnostic criteria[50,51]);

- **No clear indication for aspirin:** The participant has no diagnosed occlusive arterial disease (i.e. a history of myocardial infarction, angina pectoris, coronary or non-coronary revascularisation procedure [i.e. peripheral arterial bypass surgery or angioplasty], stroke or transient ischaemic attack);

- **No clear contra-indication to aspirin:** The participant is not at high risk of bleeding due to: gastrointestinal haemorrhage or peptic ulcer within the previous 6 months; active hepatic disease such as cirrhosis or active hepatitis; use of warfarin, or other anti-coagulant therapy; or has a history of aspirin allergy;

- **Substantial uncertainty about whether antiplatelet or omega-3 FA therapy confers worthwhile benefit:** Neither the participant nor the participant's own doctor considers there to be a definite need for the patient to take aspirin or omega-3 FA supplements regularly (or a definite need not to do so);

- **No other predominant life-threatening medical problem:** The participant does not have some condition (other than diabetes) that might limit compliance with 5 years of study treatment, such as cancer (other than non-melanoma skin cancer).

3.2 Identification of participants

3.2.1 Large numbers of potentially eligible patients can be identified through diabetes registers, trial databases and general practice

Based on our previous experience, large numbers of potentially suitable individuals may need to be approached to randomise at least 15,000 eligible patients into this long-term trial. People with diabetes will be sought from 3 main sources: diabetes registers, trial databases and general practice. Diabetologists from around the UK will be invited to collaborate and allow invitation of potentially suitable individuals from locally held diabetes registers (such as those held for retinopathy screening or for service provision). Such registers vary in size from a few thousand to many thousands and at least one third of participants are expected to be recruited from these sources. Other people with diabetes will be identified from among the populations taking part in HPS and other diabetes trials. In order to streamline the invitation process, the contact details of potentially eligible people will be sought electronically whenever possible to allow central mailings in the name of the local doctor. This approach has allowed large numbers to be recruited by CTSU into the HPS and SEARCH trials, and is more efficient and cost-effective than mailings sent from individual centres or practices. It also allows over-selection of certain groups (e.g. older individuals) to ensure an appropriate balance of different types of participant. The third source will be directly from general practice. Diabetologists and other collaborators will be asked to identify 20-30 local general practices with computerized diabetes registers, and to seek their agreement to mailing a single batch of letters to potentially eligible individuals. Experience of screening notes in general practice indicates that ~3.5% of patients aged 50-65 have diabetes without diagnosed arterial disease. Hence, a typical group practice of

about 10,000 registered patients may have 100-150 potentially eligible individuals. To complement these 3 main methods of recruitment, hospital collaborators, general practitioners or practice nurses will also be able to offer a standard “invitation pack” (containing patient information leaflet, screening questionnaire and freepost envelope) to potentially eligible participants when they are seen for routine care in their clinic, or directly by mail if they have previously agreed to be approached for research. In addition, randomised participants will have the option to recommend any friend or relative they think may be eligible and interested in participating in the study and potential participants may volunteer themselves if they hear about the study from any source.

3.3 Screening (- 2 months)

3.3.1 Establishing eligibility

Patients with diabetes that are identified from any source as being possibly suitable will be invited by letter to take part. An invitation letter will be sent enclosing an information leaflet (Appendix 1) and a brief one-page Screening questionnaire to determine eligibility and to seek consent (Appendix 2), along with a Freepost envelope. Preliminary eligibility for the pre-randomisation Run-in phase will be based on information provided on the completed Screening questionnaire (i.e. diagnosis of diabetes, no history of diagnosed occlusive arterial disease, no contraindication to regular aspirin and signed consent to participate).

3.3.2 Pre-randomisation Run-in treatment and optional blood and urine sampling

Eligible patients will be sent a Run-in pack of medication (containing placebo tablets and placebo capsules) and asked to take one tablet and one capsule daily for 2 months. An information sheet about the medication will be provided and a copy of their signed agreement to participate will also be sent to them. About 2-4 weeks later, participants will be sent an optional blood and urine sampling kit, and asked to take this kit to their general practice for sample collection (and for measurement of blood pressure, height and weight), with this sample then mailed to the central laboratory in the containers provided. A supplementary information leaflet is to be provided and separate consent sought for this 5-10ml blood and urine collection which will allow baseline stratification by important biochemical prognostic variables (such as blood HbA_1C, lipids and markers of renal function, and urinary albumin/creatinine ratio).

During the Run-in period, the participant's general practitioner will be informed by letter of their patient's possible involvement in the study and asked to return a form if they consider there to be any reason **not** to randomise their patient (in which case the patient would be informed of their GP’s decision and withdrawn before randomisation). Patients are to be randomised only if, at the end of the Run-in period, they seem likely to comply with the study protocol for several more years. By this process, many potential drop-outs should be excluded before becoming part of the randomised comparison, with a consequent improvement in statistical sensitivity of the “intention-to-treat” analyses.[52] Patients who are not eligible will be thanked for completing the questionnaire, but will not proceed further.

3.4 Randomisation (0 months)

3.4.1 Final check of eligibility and compliance before randomisation

About 2 months after they have been sent their Run-in pack, participants will be sent a further more detailed Randomisation questionnaire asking about any significant problems (including any cardiovascular events) and their compliance with the study treatments during the Run-in period. Details of their diabetes history (in particular to allow classification as type 1 or 2), current medication, ethnic group, and smoking history will be sought to allow baseline risk

stratification.[59] Participants will be asked to reiterate their commitment to a 7-year study and also, if willing, to provide details of a friend or relative living at a different address who may be contacted in the event of loss of contact with the participant.

3.4.2 Random allocation of aspirin 100mg daily versus placebo, and of 1g daily capsules containing omega-3 fatty acids versus placebo

Participants who indicate on the randomisation questionnaire that they remain eligible and willing to continue into the long-term part if the study will be randomised by the central computer in CTSU, using a minimisation algorithm to ensure balance by important baseline variables.[60] Eligible patients will be randomised in a 2 x 2 factorial blinded design between:

- Aspirin 100mg daily versus matching placebo
- Omega-3 fatty acid capsules 1 daily versus placebo

One aspirin tablet and one capsule are to be taken each day for about 7 years unless some clear reason to stop develops.

They will then be mailed a pack containing a 24-week supply of their allocated study treatment, along with relevant information about the medication and the CTSU Freefone number for any trial-related queries. The patient's general practitioner will be informed by letter of their patient's randomisation into the trial and the results of any relevant blood tests taken during Run-in (e.g. lipid profile and HbA_1C).

3.5 Post-randomisation Follow-up

3.5.1 6-Monthly follow-up questionnaires sent by mail (with telephone back-up)

Follow-up questionnaires asking about cardiovascular events, other serious adverse events (including bleeding episodes), compliance with study treatment and use of relevant non-study treatments will be sent 6-monthly with a further supply of the participant’s allocated study treatment. All randomised patients - irrespective of whether or not they continue to take study treatments - are to be encouraged to return their questionnaire with up to 2 mailed reminders sent routinely. Failure to return a questionnaire will result in a study administrator telephoning the patient in order to complete the Follow-up questionnaire. Those who do not agree to being contacted in this way will be followed via their GPs and central registries.

3.5.2 Modifying study treatment

The aspirin component of the study treatment will be discontinued if a patient starts to use regular non-study aspirin or warfarin or is considered to have developed some other clear contraindication to the study aspirin (e.g. high risk of bleeding or aspirin allergy).
(N.B. Patients who stop the aspirin component of the study will be encouraged to continue the omega-3 FA component, unless this is thought to be clearly contraindicated.)

The study treatments will also be stopped if a serious adverse experience believed with a reasonable probability to be due to study treatment is reported (see Section 3.6). Patients may also stop either study treatment at their own request, or at the request of their own doctors. But, any patient who stops the study medications would still be encouraged to continue returning their Follow-up questionnaires and, if appropriate, to continue taking either study treatment alone if the other is to be stopped.

3.5.3 Follow-up of deaths and of non-fatal cancers through central registries

All randomised patients will be flagged through the Office for National Statistics and other central registries for death, cancer and other relevant events. Consequently, unbiased cause-specific mortality and site-specific cancer incidence data for all patients can be obtained,

independent of whether they are still complying with study medication or responding to questionnaires.

3.6 Reporting serious adverse events

3.6.1 Immediate reporting of expected and unexpected serious adverse events believed with a reasonable probability to be due to study treatment

To fulfil regulatory authority requirements, serious adverse events believed with a reasonable probability to be due to study treatment are to be reported immediately by telephoning the 24-hour Freefone service, where a few brief details will be recorded. For the purposes of this study, the only adverse events that need to be reported in this way are those that are **both**:

- (i) serious (defined as any untoward medical occurrence which results in death is life-threatening, requires hospitalisation or the prolongation of existing hospitalisation, results in persistent or significant disability or incapacity, congenital abnormality, or the result of an overdose); **and**
- (ii) believed with a reasonable probability to be due to study treatment.

All such serious drug related adverse events (whether expected or not) will be reported (unblinded) to the Chairman of the independent Data Monitoring Committee, and included in the Annual Safety Report for the Research Ethics Committee, to Bayer Healthcare AG and to Abbott Products Operations AG (formerly Solvay Pharmaceuticals). Any such serious drug related adverse events which are unexpected (SUSARs) will be reported, unblinded in an expedited fashion to the Medicines and Healthcare products Regulatory Agency (MHRA) and to the companies. Expected aspirin related serious adverse events might include those due to bleeding, gastro-intestinal perforation, broncho-spasm or other recognised side-effects of aspirin; there are no expected omega-3 fatty acid related serious adverse events.

3.6.2 Reporting of other serious adverse events on routine follow-up questionnaires

Any serious adverse events that are not thought to be due to study treatment, including study endpoints, should not be reported in this way. Such events are, however, to be routinely recorded on the Follow-up questionnaires (see Section 3.5) for central analysis and regular review by the Data Monitoring Committee (see Section 2.3).

3.6.3 Unblinding of study treatment allocation

Unblinding of study treatment allocation is available via the 24-hour Freefone service, where all such unblindings are logged. In general, unblinding of patients is only likely to be necessary if knowledge of treatment allocation will influence immediate patient management or for onward reporting of serious drug related adverse events (see Section 3.6.1).

3.7 Central ascertainment of biochemical effects and confirmation of reported vascular events, cancers and death

3.7.1 Assessing biochemical efficacy of study treatments by random sampling

As well as asking all participants routinely about their compliance with allocated study treatments, compliance will be assessed in a random sample of participants at intervals during the study. A randomly selected sub-set of randomised participants (5-10%) will be sent a kit for blood collection by their GP and mailing to the coordinating centre. Assays of serum or urine thromboxane levels to assess aspirin effects[61] and blood markers of omega-3 FA intake,[62] will be measured to estimate compliance with study treatments. At least once during follow-up assessments will be made in a random sample of participants of the effects of study treatments on blood HbA1c, lipids and markers of renal function, and on urinary albumin/creatinine ratio.

3.7.2 Confirmation of patient reported cardiovascular and other significant serious adverse events using mail-based systems

The coordinating centre will seek confirmation and additional information (including, if necessary, any relevant hospital discharge records) from the participant's GPs about each suspected myocardial infarction, stroke, coronary or non-coronary angioplasty, arterial surgery, cancer, or other relevant hospitalisation or serious adverse event recorded on Follow-up questionnaires or reported by participants during telephone calls or other contact. Similarly, further information will be sought from participant's GPs and other relevant sources about all cancers and deaths identified from national registries. All such information will then be reviewed, blind to treatment allocation, by coordinating centre clinical staff and coded in accordance with pre-specified criteria. The diagnosis of myocardial infarction (MI) requires information about either: (i) the presence of two or more of: (a) typical ischaemic chest pain, pulmonary oedema, syncope or shock; (b) development of pathological Q-waves and/or appearance or disappearance of localised ST-elevation followed by T-wave inversion in two or more of twelve standard electrocardiograph leads; and (c) increase in concentration of biochemical markers consistent with MI (e.g. CK >2xULN, or elevated troponins); or (ii) necropsy findings of MI of an age corresponding to time of onset of symptoms. (Silent myocardial infarctions are not to be included.) Stroke is defined as rapid (or uncertain) onset of focal or global neurological deficit lasting >24 hours or leading to death and transient ischaemic attack is defined by the same symptoms lasting <24 hours. Information (e.g. CT/MRI scan results) will be sought to ascertain the likely aetiology of the stroke (i.e. haemorrhagic or not). These procedures for reviewing reports from patients and other sources of possible study outcomes was developed by CTSU for the MRC/BHF Heart Protection Study, and allowed over 98% of such reports to be successfully confirmed or refuted.

Appendix 1: Information leaflet for potentially eligible patients

ASCEND: Patient Information Leaflet [V8.4_121010]

ASCEND: Invitation to join a large medical research project

A randomised study of aspirin and of natural oils for the primary prevention of cardiovascular events in diabetes

You are being invited to take part in a research study. Before you decide whether to participate, it is important for you to understand why the research is being done and what is involved. Please take time to read the following information carefully and discuss it with friends or relatives if you wish. You are entirely free to decide whether or not to take part in this trial. If you choose not to take part, the standard of care you are given by your own doctors will not be affected. If there is anything that is not clear, or if you would like more information, please telephone the ASCEND Freefone number (0800 585323) and speak to a study nurse or doctor. The study is to be conducted mainly by mail, so no extra clinic visits will be required.

Aspirin, heart disease and strokes

Patients with diabetes may be at increased risk of developing heart disease or suffering a stroke. Aspirin prevents heart attacks and strokes in people who have existing problems with their heart or blood circulation. But it is not known whether aspirin would be helpful in people with diabetes who have not yet been diagnosed with heart or circulatory problems. Serious (but uncommon) complications from the regular use of aspirin are bleeding in the stomach or intestinal tract. Typically this might happen in only about 1 per 1000 people taking aspirin regularly each year. Extremely rarely, aspirin may cause bleeding in the brain (about 1-2 per 10,000 people taking aspirin each year). Previous studies in people with known circulatory problems have shown that about 10 times as many people given aspirin have avoided a heart attack or stroke as have experienced a serious complication. However, in people with diabetes and no circulatory problems, it is not known whether the benefits of aspirin will outweigh the possible risks.

Omega-3 fatty acids and diabetes

Naturally occurring oils that are rich in omega-3 fatty acids (such as fish oils) may reduce the chances of a recurrent heart attack among people who have survived at least one heart attack. These oils have not been widely tested in people with diabetes, but there are reasons to hope that they may be helpful (although this is unproven). Taking regular supplements of such oils may have little or no beneficial effect among people living in a country (such as Britain) where most people eat a balanced diet. It is also possible that the long term use of these oils could, on balance, be slightly harmful – but this too is unknown.

What the study hopes to answer

The main purpose of the ASCEND study, is to find out whether long-term treatment with aspirin in people with diabetes who are not already known to have heart or circulatory problems, produces benefits by preventing heart attacks and strokes which outweigh the possible risks of bleeding. ASCEND will also help establish whether oils rich in omega-3 fatty acids are of any importance in reducing the chances of having a heart attack in people with diabetes who have not yet got circulatory problems.

Why have I been chosen?

ASCEND will involve at least ten thousand men and women from around Britain, who like you, are being invited to take part because they have diabetes. This invitation has come from either your own GP or a local Specialist because they think you might be suitable for the study. Alternatively you may have been recommended by a friend or relative who is already taking part in the study or volunteered yourself having read about the study. It is up to you to decide whether or not to take part in this study.

If you do decide to take part, you would, of course, be free to withdraw from the study treatment at any time without necessarily giving any reason (and without adversely affecting the medical care you can expect from your own doctors). In particular, at the end of the first 2 months, when you finish your first box of treatment, you will have the chance to withdraw if you have any second thoughts or problems with study treatment.

What taking part in ASCEND involves

Everyone taking part will have agreed to do so voluntarily, knowing that it may involve them in taking study treatment for at least 5 years. The daily study treatments (which would be sent to you by mail) will be a single white tablet and a single brown capsule taken from a blister pack. The white tablets will contain either active aspirin (100mg) or a similar looking inactive substance called a “placebo”. Whether or not a participant receives active or placebo tablets will be determined randomly (like tossing a coin). Each participant will have a 50% chance of receiving active aspirin and a 50% chance of receiving placebo (“dummy”) tablets. The brown capsules will each contain 1 gram of a naturally occurring oil, either mainly omega-3 fatty acids or mainly olive oil. Each participant will have a 50% chance of receiving the omega-3 containing capsules and a 50% chance of receiving olive oil capsules. The type of study treatment being taken will not generally be known by you or your doctor. This information will be known only by certain staff at the coordinating centre in Oxford, but it would be made available to your doctor if this were ever medically necessary. This design helps ensure that reliable information will be obtained about the effects of these potentially important treatments.

What you have to do to join the study

If you might like to participate in this study you should complete the brief Screening Questionnaire on the inside of the letter, sign the Agreement to Participate and return them both in the enclosed Freepost envelope. We will use your answers on the questionnaire to check that you are suitable for the study. If you are suitable, then we will send a box of conveniently packaged study treatments, and ask you to start taking one tablet and one capsule each day by mouth for the next 2 months. We shall also inform your general practitioner of your involvement in the study and check that they are happy for you to continue in the study.

Within a few weeks of receiving this first pack of study treatment, you will also be sent an **optional** blood and urine sampling kit. If it is convenient for you to do so, you would be asked to attend your local surgery to have a small blood sample taken (about 2 teaspoons full) and to provide a urine specimen. Measurements of your height, weight and blood pressure would also be recorded at the surgery and this information, along with the sample, would then be mailed to the ASCEND coordinating centre.

Long-term commitment to the study

Towards the end of the 2 months you will be sent a second study questionnaire. This will allow you to indicate whether or not you would be willing to continue taking the study treatments long-term. Participation in the study does require a commitment to take the study treatments regularly for at least 5 years and to complete questionnaires regularly. **If you do not think that you would be willing or able to do this then it would be better not to join in the first place.**

If you decide to continue you would then be sent further supplies of the study treatments and asked to take one tablet (which would be active or dummy aspirin) and one capsule (containing one or other naturally-occurring oil) every day for the next 5 years. Further questionnaires would be sent out at 6-monthly intervals. We would ask you to tell us about your current medication and any changes to your health since your last questionnaire. Additional supplies of study treatment would be sent to you 6-monthly if you were willing to continue taking it. If you do stop during the first 2 months then no further enquiries will be made of you. But, if you decide to continue, we would like to remain in contact with you for the next several years – even if you stop taking the study treatment during this period. **Throughout the study, your own doctors would remain fully responsible for all your other medical care as usual.** However, if you develop any unexpected symptoms which you believe

may be due to study treatment you should contact a study doctor on the 24-hour Freefone service: 0800 585323.

What are the side-effects and risks of taking part?

A low dose of aspirin is being used in this study in order to minimise any stomach upset or other gastrointestinal problems. Some minor bleeding (e.g. after having blood taken) and bruising may be experienced by some people, but serious bleeding is likely to be rare. We shall monitor whether aspirin causes an unacceptable level of bleeding during the study. Bleeding risks with aspirin may be somewhat greater among those who are taking warfarin (Marevan) or other blood thinning drugs (e.g. Acenocoumarol (Nicoumalone, Sinthrome) or Phenindione). So, if you are taking any of these blood thinning drugs you would not be suitable to join the study, and if you are prescribed them later we recommend stopping the study aspirin/placebo tablets. People who join the study would be asked to avoid taking aspirin-containing painkillers, and to take an alternative, (such as paracetamol), whenever pain relief is necessary. All other prescribed treatments can be taken as usual. There are no other lifestyle or dietary restrictions required. The doses of the naturally occurring oils being tested in ASCEND are not known to cause any particular problems, although some people may experience gastro-intestinal ("tummy") disturbances. If you did experience any symptoms that you thought were related to either of the study treatments, medical advice is available at all times through the 24-hour Freefone service: 0800 585323.

What are the possible benefits of taking part?

We hope that both the study treatments may help you. However, this cannot be guaranteed. The information we get from this study may help us to treat future patients with diabetes better and may help to prevent many thousands of heart attacks and strokes.

What if new information becomes available?

Sometimes during the course of a research project, relevant new information becomes available about the treatment that is being studied. If this happens we will tell you and your general practitioner about it and you can discuss whether you want to continue in the study. A study doctor is available through the 24-hour Freefone service if either you or your GP need to discuss any new information.

What happens at the end of the study?

When the research study finishes, we will inform you and your GP of the study results. You will then be able to decide whether or not you should take aspirin and/or omega-3 fatty acids. After the study finishes we will no longer continue to provide study medication for you. But, if the study results suggest possible benefit, you could discuss with your GP whether you should take either of these treatments routinely. We will also publish the study results in a professional medical journal as soon as possible after the study finishes. You would not be identified individually in any published report.

What if something goes wrong?

In the unlikely event of you being harmed as a result of taking part in the ASCEND study, the University of Oxford provides insurance cover and you would retain the same rights of care as any other patient treated in the National Health Service.

Will my taking part in this study be kept confidential?

The coordinating centre would seek information from participants' own doctors and from NHS and other central registries about any serious illnesses (such as heart attacks, strokes, cancers etc) that occur. All such information would be used, in confidence, only for medical research purposes and for routine regulatory and audit purposes.

Study organisation

The ASCEND study has been designed, and is coordinated, by Oxford University's Clinical Trial Service Unit. It involves the collaboration of many doctors and nurses around the country. The study design has been reviewed and agreed by independent Research Ethics Committees, which include people from outside the medical profession. The British Heart Foundation has provided a grant to

conduct this research study, and packaged study treatment has been provided free by Bayer (makers of the aspirin/placebo) and Abbott (who are providing the natural oils). An independent Data Monitoring Committee will review various outcomes among participants during the study, and will inform the organisers if any important new information has emerged that needs to be provided to participants and their doctors. Any questions about the study should be directed to the coordinating centre in Oxford either by telephone (24-hour Freefone service: 0800 585323) or by mail to: ASCEND Study, CTSU, Richard Doll Building, University of Oxford, Old Road Campus, Oxford, OX3 7LF. Alternatively you can e-mail us on ascend@ctsu.ox.ac.uk.

ASCEND: Summary of invitation to join a large medical research project

- Having diabetes may increase the risk of heart attacks and strokes
- Aspirin and omega-3 fatty acids benefit people who have survived a heart attack
- It is not clear whether people with diabetes who have not shown signs of circulatory problems should take aspirin or omega-3 supplements regularly
- Most people with diabetes and no circulatory problems do not take aspirin or omega-3 supplements regularly
- Low-dose aspirin is generally very safe, but does increase the risk of bleeding
- Omega-3 fatty acids at the doses being taken in ASCEND are also considered safe
- The purpose of ASCEND is to find out whether aspirin and/or omega-3 fatty acid supplementation prevents heart attacks and strokes in people with diabetes who have not shown signs of circulatory problems
- If these treatments are shown to be safe and effective for people with diabetes, then their widespread use could lead to the prevention of many thousands of heart attacks and strokes and the saving of many lives
- With your help we can answer these questions reliably with the ASCEND study

If you have any questions about the study then please feel free to contact the coordinating centre on Freefone: 0800 585323

If you think you might be interested in joining this research study please complete and return the attached questionnaire and agreement to participate. A copy of your signed agreement to participate will be returned to you when your first pack of study treatment is sent out.

Please keep this information sheet for your own records.

THANK YOU FOR YOUR HELP

Appendix 2: Consent form

Need help completing this form? Please call Freefone 0800 585323

Please read this **Agreement to Participate**, and if you are willing then please CROSS the boxes, SIGN and DATE the form using blue or black ink, and return it in the FREEPOST envelope provided.

7. Agreement to Participate

Please cross (☒) **EVERY box** to confirm that you have read and understood the following:

☐	I have read and understood the leaflet **"ASCEND: Invitation to join a large medical research project"** *[Version number of accompanying Patient Information Leaflet will be inserted here]*
☐	I have had an opportunity to telephone the Freefone number 0800 585323 and ask any relevant questions. All my questions have been answered to my satisfaction **OR** I decided that I did not need to ask any questions
☐	I understand that my participation in the ASCEND study is voluntary and that I am free to withdraw from the study at any time without my medical care or rights being affected
☐	I understand that information about my progress in the ASCEND study will be recorded on a computer database, and that these data will be stored securely and confidentially on a computer at Oxford University
☐	I agree that information about any serious illnesses (such as heart attacks, strokes or cancers) may be supplied in confidence to the study coordinators by my own doctors and by NHS and other central registries for use in the ASCEND study
☐	I agree that my hospital and other health records may be looked at in confidence by authorised individuals from the ASCEND study and by regulatory authorities to check the study is being carried out correctly
☐	I understand that my GP will be informed about this provisional agreement to participate in the ASCEND study, and that in about 2 months time I will have another opportunity to decide whether or not I want to join the long-term part of the study

I am happy to take part in ASCEND:

ASCEND Screening Questionnaire [V3.4_240407]

Signature: ____________ (Please use blue or black ink)

& **PRINTED** name: ____________ Today's date: __ __ / __ __ / 2 0 __ __
Day Month Year

Please check that you have answered every question, and signed and dated the form. Return the completed form in the Freepost envelope provided (no stamps needed) to:-

Freepost RLUJ-TKES-SURB, ASCEND, Richard Doll Building, University of Oxford, Old Road Campus, Headington, Oxford, OX3 7LF

If you have any questions about the study, please contact the coordinating centre in Oxford on FREEFONE: 0800 585323 (preferably during office hours 9 am - 5 pm, Monday to Friday)

If this questionnaire indicates that you are suitable to enter the preliminary part of ASCEND, a box containing ASCEND tablets (aspirin or placebo) and capsules (one or other natural oil) will be mailed to you. A copy of this Agreement to Participate, for you to keep, will also be mailed.

If the questionnaire suggests that the study medications may not be suitable for you, then we shall write and tell you.

Thank you very much

4

References:

1. Murray C, Lopez A. Global Health Statistics. World Health Organisation 1996.
2. King H, Aubert RE, Herman WH. Global burden of diabetes, 1995-2025: Prevalence, numerical estimates, and projections. Diabetes Care 1998;21:1414–31
3. Pyörälä K, Laakso M, Uusitupa M. Diabetes and atherosclerosis: an epidemiologic view. Diabetes/Metabolism Reviews 1987;3:463-524
4. Panzram G. Mortality and survival in type two (non-insulin-dependent) diabetes mellitus. Diabetologia 1987;30:123-131
5. Rolka DB, Fagot-Campagna A, Narayan KM. Aspirin use among adults with diabetes. Diabetes Care 2001;24:197-201
6. UK Prospective Diabetes Study Group: UK Prospective Diabetes Study 6: complications in newly diagnosed type 2 diabetic patients and their association with different clinical and biochemical risk factors. Diabetes Res 1990:13;1-11
7. Antiplatelet Trialists' Collaboration. Collaborative overview of randomised trials of antiplatelet therapy. I: Prevention of death, myocardial infarction, and stroke by prolonged antiplatelet therapy in various categories of patients. BMJ 1994;308:81-106
8. Antithrombotic Trialists' Collaborative Group. Collaborative meta-analysis of randomised trials of antiplatelet therapy for prevention of death, myocardial infarction and stroke in high risk patients. BMJ 2002;324:71-86
9. EUROASPIRE Study Group. EUROASPIRE. A European Society of Cardiology survey of secondary prevention of coronary heart disease: principal results. Eur Heart J 1997;18:1569-1582
10. EUROASPIRE I and II Group. Clinical reality of coronary prevention guidelines: a comparison of EUROASPIRE I and II in nine countries. Lancet 2001;357:995-1001
11. British Cardiac Society, British Hyperlipidaemia Association, British Hypertension Society, endorsed by the British Diabetic Association. Joint British recommendations on prevention of coronary heart disease in clinical practice. Heart 1998;80:S1-S9
12. Wood DA, De Backer G, Faergeman O et al. Prevention of coronary heart disease in clinical practice. Recommendations of the Second Joint Taskforce of the European Society of Cardiology, European Atherosclerosis Society and European Society of Hypertension. Eur Heart J 1998;19:1434-1503
13. MRC/BHF Heart Protection Study Collaborative Group. MRC/BHF Heart Protection Study of cholesterol lowering with simvastatin in 20 536 high-risk individuals. Lancet 2002;360:7-22
14. UK Prospective Diabetes Study Group. Tight blood pressure control and risk of macrovascular and microvascular complications in type 2 diabetes: UKPDS 38. BMJ 1998;317:703-13
15. ETDRS Investigators. Aspirin effects on mortality and morbidity in patients with diabetes mellitus. JAMA 1992;268:1292-1300
16. American Diabetes Association Position Statement. Aspirin therapy in diabetes. Diabetes Care 2001;21:S45-46
17. Hippisley-Cox J, Pringle M. General practice workload implications of the national service framework for coronary heart disease: cross sectional survey. BMJ 2001;323:269-70
18. Colwell JA. Aspirin therapy in diabetes is underutilized. Diabetes Care 2001;24:195-198
19. MRC/BHF Heart Protection Study Collaborative Group. MRC/BHF Heart Protection Study of cholesterol-lowering therapy and of antioxidant vitamin supplementation in a wide range of patients at increased risk of coronary heart disease death: early safety and efficacy experience. Eur Heart J 1999;20:725-741
20. Current Controlled Trials metaRegister (Accessed Sept 02) The prevention of progression of asymptomatic diabetic arterial disease (POPADAD).
21. Patrono C. Aspirin as an Antiplatelet drug. N Engl J Med 1994;330:1287-1294
22. Kelly JP, Kaufman DW, Jurgelon JM, Sheehan J, Koff RS, Shapiro S. Risk of aspirin-associated major upper-gastrointestinal bleeding with enteric-coated or buffered product. Lancet 1996;348:1413-6
23. Kris-Etherton PM, Harris WS, Appel LJ for the Nutrition Committee. Fish consumption, fish oil, Omega-3 fatty acids, and cardiovascular disease. Circulation 2002;106:2757
24. He K, Rimm EB, Merchant A, et al. Fish consumption and risk of stroke in men. JAMA 2002;288: 3130-3136.
25. Bang HO, Dyerberg J, Neilsen AB. Plasma lipid and lipoprotein patterns in Greenland west-coast Eskimos. Lancet 1971;(i):1143-44

26. Marckmann P, Grønbæk M. Fish consumption and coronary heart disease mortality. A systematic review of prospective cohort studies. Eur J Clin Nutr 1999;53:585-590
27. Hu FB, Bronner L, Willett WC, et al. Fish and Omega-3 fatty acid intake and risk of coronary heart disease in women. JAMA 2002;287:1815-1821
28. Albert CM, Campos H, Stampfer MJ, et al. Blood levels of long-chain n-3 fatty acids and the risk of sudden death. N Eng J Med 2002;346(15):1113-1118
29. Siscovick DS, Raghunathan TE, King I, et al. Dietary intake of long-chain n-3 polyunsaturated fatty acids and the risk of primary cardiac arrest. Am J Clin Nutr 2000; 71(1 suppl):208S-212S
30. Hu FB, Cho E, Rexrode KM, Albert CM, Manson JE. Fish and long-chain ω-3 fatty-acid intake and risk of coronary heart disase and total mortality in diabetic women. Circulation 2003;107:1852-7
31. GISSI-Prevenzione Investigators. Dietary supplementation with n-3 polyunsaturated fatty acids and vitamin E after myocardial infarction: results of the GISSI-Prevenzione trial. Lancet 1999;354:447-55
32. Burr ML, Gilbert JF, Holliday RM, et al. Effects of changes in fat, fish and fibre intakes on death and myocardial reinfarction: Diet and Reinfarction Trial (DART). Lancet 1989;ii:757-761
33. Bucher HC, Hengstler P, Schindler C, Meier G. N-3 Polyunsaturated fatty acids in coronary heart disease: A Meta-analysis of randomized controlled trials. Am J Med 2002;112:298-304
34. Durrington PN, Bhatnagar D, Mackness MI, Morgan J, Julier K, Khan MA, France M. An omega-3 polyunsaturated fatty acid concentrate administered for one year decreased triglycerides in simvastatin treated patients with coronary heart disease. Heart 2001;85:544-548
35. Force T, Milani R, Hibberd P, Lorenz R, Uedelhoven W, Leaf A, Weber P. Aspirin-induced decline in prostacyclin in patients with coronary artery disease is due to decreased endoperoxide shift. Circulation 1991;84:2286-2293
36. Connor WE. Importance of n-3 fatty acids in health and disease. Am J Clin Nutr 2000;71 (suppl):171S-5S
37. Thies F, Garry JMC, Yaqoob P, Rerkasm K, Williams J et al. Association of n-3 polyunsaturated fatty acids with stability of atherosclerotic plaques: a randomised controlled trial. Lancet 2003;361:477-85
38. Geleijnse JM, Giltay EJ, Grobbee DE, Donders DE, Kok FJ. Blood pressure response to fish oil supplementation: metaregression analysis of randomised trials. J Hypertension 2002;20(8):1493-9
39. Harris WS. N-3 Fatty acids and serum lipoproteins: human studies Am J Clin Nutr.1997;65(5 Suppl):1645S-16454S
40. Sitori CR, Paoletti R, Mancini M, Crepaldi G, Manzato E, Rivellese A et al. n-3 Fatty acids do not lead to an increased diabetic risk in patients with hyperlipidaemia and abnormal glucose tolerance. Am J Clin Nutr 1997;65:1874-81
41. Roche HM, Gibney MJ. Postprandial triacylglycerolaemia: the effect of low-fat dietary treatment with and without fish oil supplementation. Eur J Clin Nutr 1996;50:617-624.
42. Davi G, Catalano I, Averna M, Notarbartolo A, Strano A, Ciabattoni G, Patrono C. Thromoboxane biosynthesis and platelet function in type II diabetes mellitus. N Engl J Med 1990;322:1769-74
43. Farmer A, Montori V, Dinneen D, Clar C. Fish oil in people with type 2 diabetes mellitus (Cochrane Review). The Cochrane Library, Issue 2, 2002. Oxford: Update Software
44. Peto R, Gray R, Collins R, Wheatley K, Hennekens C, Jamrozik K, Warlow C, Hafnet B, Thompson E, Norton S, Gilliland J, Doll R. Randomised trial of prophylactic aspirin in British male doctors. BMJ 1988;296:313-316
45. Physicians' Health Study Research Group. Final Report of the aspirin component of the ongoing Physicians' Health Study1989. N Engl J Med 1989;321:129-35
46. Rexrode KM, Lee IM, Cook NR et al. Baseline characteristics of participants in the Women's Health Study. J Women's Health Gend-based Med 2000;9:19-27
47. Christen WG, Gaziano JM, Hennekens CH. Design of Physician's Health Study II, a randomized trial of beta-carotene, vitamins E and C and multivitamins, in prevention of cancer, cardiovascular disease, and eye disease, and review of completed trials. Ann Epidemiol 2000;10(2):125-34
48. Buring J. (personal communication) Women's Antioxidant Cardiovascular Study 2001
49. Liu S, Manson JE, Lee I-M, Cole S, Hennekens CH, Willett WC, Buring J. Fruit and vegetable intake and risk of cardiovascular disease: the Women's Health Study. Am J Clin Nutr 2000;72:922-8
50. The Expert Committee on the Diagnosis and Classification of Diabetes Mellitus. Report of the Expert Committee on the Diagnosis and Classification of Diabetes Mellitus. Diabetes Care 1997;20:1183-97
51. Alberti KGMM, Zimmet PZ for the WHO Consultation. Definition, diagnosis and classification of diabetes mellitus and its complications. Part 1: diagnosis and classification of diabetes mellitus. Provisional report of a WHO consultation. Diabetes Care 1998;15:539-53

52. Lang JM, Buring JE, Rosner B, Cook C, Hennekens CH. Estimating the effect of the Run-in on the power of the Physicians' Health Study. Stat Med 1991;10:1585-93
53. Peto R, Pike MC, Armitage P, et al. Design and analysis of randomized clinical trials requiring prolonged observation of each patient. Part II: Analysis and examples. Br J Cancer 1977;35:1-39
54. Peto R, Pike MC, Armitage P, et al. Design and analysis of randomized clinical trials requiring prolonged observation of each patient. Part I: Introduction and design. Br J Cancer 1976;34:585-612
55. Haffner SM, Lehto S, Rönnemaa T, Pyörälä K, Laakso M. Mortality from coronary heart disease in subjects with type 2 diabetes and in nondiabetic subjects with and without prior myocardial infarction. N Engl J Med 1998;339:229-234
56. UK Prospective Diabetes Study (UKPDS) Group. Intensive blood-glucose control with sulphonylureas or insulin compared with conventional treatment and risk of complications in patients with type 2 diabetes (UKPDS 33). Lancet 1998;352:837-853
57. MRC/BHF Heart Protection Study Collaborative Group. MRC/BHF Heart Protection Study of cholesterol lowering with simvastatin in 5963 people with diabetes. Lancet 2003;361:2005-2016
58. Baigent C, Blackwell L, Collins R, Emberson J, Godwin J, Peto R, et al. Aspirin in the primary and secondary prevention of vascular disease: collaborative meta-analysis of individual participant data from randomised trials. Lancet. 2009;373:1849-60.
59. Prior MJ, Prout T, Miller D et al and the ETDRS Research Group. C-peptide and the classification of diabetes mellitus in the early treatment of diabetic retinopathy study. Report number 6. Ann Epidemiol 1993;3:9-17
60. Pocock SJ, Simon R. Sequential treatment assignment with balancing for prognostic factors in the controlled clinical trial. Biometrics 1975;31:103-115
61. Lawson JA, Brash AR, Doran J, Fitzgerald GA. Measurement of urinary 2,3-dinor-thromboxane B_2 and thromboxane B_2 using bonded-phase phenylboronic acid columns and capillary gas chromatography-negative ion-chemical ionization mass spectrometry. Anal Bioch 1985;150:463-70
62. Zelniuch-Jacquotte A, Chajes V, Van Kappel AL, Riboli E, Toniolo P. Reliability of fatty acid composition in human serum phospholipids. Eur J Clin Nutr 2000;54:367-72

ASCEND: STUDY ORGANISATION

STEERING COMMITTEE
(Major organisational and policy decisions)

Study coordinator	J Armitage
Clinical coordinator	L Bowman
Chairman of Steering Committee	R Collins
Statisticians	S Parish
	R Peto
Administrative coordinator	J Barton
Lay member	D Simpson
Other members	A Adler
	C Baigent
	J Bodansky
	A Farmer
	HAW Neil
	P Sleight
	N Samani
	R Haynes
	R McPherson
	M Mafham

DATA MONITORING COMMITTEE
(Interim analyses and response to specific concerns)

Chairman	P Sandercock
Other members	R Gray, C Hennekens, H Gerstein

CENTRAL COORDINATION
CLINICAL TRIAL SERVICE UNIT (CTSU), OXFORD

Administrative coordinators	Ms Jill Barton, Mr Kevin Murphy
Clinical coordinators	Dr Jane Armitage, Dr Louise Bowman
Computing coordinators	Dr A Young, Dr Mike Lay

ASCEND Study Office
CTSU
Richard Doll Building
University of Oxford
Old Road Campus
Headington
Oxford, OX3 7LF
Office telephone: +44 (0) 1865 743888
Office fax: +44 (0) 1865 743981
