# Supplementary material for: Comparison of the Accuracy and Completeness of Records of Serious Vascular Events in Routinely Collected Data vs Clinical Trial–Adjudicated Direct Follow-up Data in the UK: Secondary Analysis of the ASCEND Randomized Clinical Trial
Source: JAMA Netw Open. 2021 Dec 28;4(12):e2139748. doi: 10.1001/jamanetworkopen.2021.39748 (PMC8715347; doi:10.1001/jamanetworkopen.2021.39748)
Supplement: Supplement 2. — eTable 1. Codes Used to Define Any Serious Vascular Event or Revascularization in Routine Data eTable 2. Baseline Characteristics of the ASCEND Trial Population eTable 3. Agreement of Routine Data vs Adjudicated Direct Follow-up for Any Serious Vascular Event Including Transient Ischemic Attack, by Subgroups eTable 4. Agreement of Routine Data vs Adjudicated Direct Follow-up, by Diagnostic Position eTable 5. Comparison of Routine Data Event Date vs Adjudicated Direct Follow-up eTable 6. Information From Adjudicated Direct Follow-up for Events Only Considered to Be Serious Vascular Events in Routine Data eTable 7. Information From Routine Data for Events Only Considered to Be Serious Vascular Events in Adjudicated Direct Follow-up eTable 8. Agreement of Routine Data vs Adjudicated Direct Follow-up, Where Adjudicated Data Restricted to Fatal and Hospitalized Event Only eTable 9. Comparison of Estimated Treatment Effect Sizes Between Adjudicated Follow-up and Routine Data for the Aspirin and ω-3 Fatty Acids Randomized Comparisons eFigure 1. Methods of Follow-up in the ASCEND Trial eFigure 2. Association of Allocation to Aspirin vs Matching Placebo and to ω-3 Fatty Acids vs Matching Placebos With Serious Vascular Event or Revascularization Using Routine Data Follow-up, Where Adjudicated Data Restricted to Fatal and Hospitalized Event Only [file jamanetwopen-e2139748-s002.pdf]

Supplementary Online Content

Harper C, Mafham M, Herrington W, et al. Comparison of the accuracy and completeness of records of serious vascular events in routinely collected data vs clinical trial–adjudicated direct follow-up data in the UK: secondary analysis of the ASCEND randomized clinical trial. *JAMA Netw Open*. 2021;4(12):e2139748. doi:10.1001/jamanetworkopen.2021.39748

eTable 1. Codes Used to Define Any Serious Vascular Event or Revascularization in Routine Data

eTable 2. Baseline Characteristics of the ASCEND Trial Population

eTable 3. Agreement of Routine Data vs Adjudicated Direct Follow-up for Any Serious Vascular Event Including Transient Ischemic Attack, by Subgroups

eTable 4. Agreement of Routine Data vs Adjudicated Direct Follow-up, by Diagnostic Position

eTable 5. Comparison of Routine Data Event Date vs Adjudicated Direct Follow-up

eTable 6. Information From Adjudicated Direct Follow-up for Events Only Considered to Be Serious Vascular Events in Routine Data

eTable 7. Information From Routine Data for Events Only Considered to Be Serious Vascular Events in Adjudicated Direct Follow-up

eTable 8. Agreement of Routine Data vs Adjudicated Direct Follow-up, Where Adjudicated Data Restricted to Fatal and Hospitalized Event Only

eTable 9. Comparison of Estimated Treatment Effect Sizes Between Adjudicated Follow-up and Routine Data for the Aspirin and ω-3 Fatty Acids Randomized Comparisons

eFigure 1. Methods of Follow-up in the ASCEND Trial

eFigure 2. Association of Allocation to Aspirin vs Matching Placebo and to ω-3 Fatty Acids vs Matching Placebos With Any Serious Vascular Event or Revascularization Using Routine Data Follow-up, Where Adjudicated Data Restricted to Fatal and Hospitalized Event Only

This supplementary material has been provided by the authors to give readers additional information about their work.

© 2021 Harper C et al. *JAMA Network Open*.

eTable 1. Codes Used to Define Any Serious Vascular Event or Revascularization in Routine Data

a) Diagnoses

Outcome	ICD-10 codes
Non-fatal myocardial infarction	Acute myocardial infarction (I21); subsequent myocardial infarction (I22)
Non-fatal presumed ischaemic stroke	Cerebral infarction (I63); stroke, not specified as haemorrhage or infarction (I64)
Vascular death excluding intracranial haemorrhage	Diseases of the circulatory system, excluding haemorrhagic stroke (I00:I52, I63:I99)
Transient ischaemic attack	Transient cerebral ischaemic attacks, excluding transient global amnesia (G45.0:G45.3, G45.8:G45.9)

b) Procedures

Outcome	OPCS-4 codes
Coronary revascularisation	Revascularisation of wall of heart (K23.4); coronary artery bypass graft (K40:K46); coronary angioplasty (K49:K50); percutaneous transluminal balloon angioplasty and insertion of stent into coronary artery (K75)
Non-coronary revascularisation (excluding amputation)	Extra-anatomic bypass of aorta (L16); emergency replacement of aneurysmal segment of aorta (L18); other replacement of aneurysmal segment of aorta (L19); other bypass of segment of aorta (L20:L21); plastic repair of aorta (L23); other open operations of aorta (L25); transluminal operations of aorta (L26.1:L26.3, L26.5:L26.9); transluminal insertion of stent graft for aneurysmal segment of aorta (L27); transluminal operations on aneurysmal segment of aorta (L28); reconstruction of carotid artery (L29); other open operations on carotid artery (L30); transluminal operations on carotid artery (L31.1, L31.3:L31.9); subclavian artery surgery (L37:L39.2, L39.5:L39.9); iliac artery surgery (L48:L54.2, L54.4:L34.9); femoral artery surgery (L56:L63.2, L63.5:L63.9); other therapeutic transluminal operations on artery (L66); repair of other artery (L68)

ICD-10 = International Classification of Diseases (tenth revision). OPCS-4 = Office of Population Censuses Surveys Classification of Surgical Operations and Procedures (fourth revision).

© 2021 Harper C et al. *JAMA Network Open*.

eTable 2. Baseline Characteristics of the ASCEND Trial Population

Characteristic	All participants	Aspirin comparison		Omega-3 fatty acids comparison	
		Treatment group	Placebo group	Treatment group	Placebo group
Number of participants	15480	7740	7740	7740	7740
Age (years)					
Mean	63.3 (9.2)	63.2 (9.2)	63.3 (9.2)	63.3 (9.2)	63.3 (9.2)
<60	5590 (36.1%)	2795 (36.1%)	2795 (36.1%)	2791 (36.1%)	2799 (36.2%)
60 to <70	6247 (40.4%)	3123 (40.3%)	3124 (40.4%)	3127 (40.4%)	3120 (40.3%)
≥70	3643 (23.5%)	1822 (23.5%)	1821 (23.5%)	1822 (23.5%)	1821 (23.5%)
Male	9684 (62.6%)	4843 (62.6%)	4841 (62.5%)	4842 (62.6%)	4842 (62.6%)
White race	14935 (96.5%)	7467 (96.5%)	7468 (96.5%)	7467 (96.5%)	7468 (96.5%)
Participant-reported hypertension	9533 (61.6%)	4766 (61.6%)	4767 (61.6%)	4768 (61.6%)	4765 (61.6%)
Duration of diabetes (years)					
Median	7 (3-13)	7 (3-13)	7 (3-13)	7 (3-12)	7 (3-13)
<9	8659 (55.9%)	4337 (56.0%)	4322 (55.8%)	4332 (56.0%)	4327 (55.9%)
≥9	5965 (38.5%)	2976 (38.4%)	2989 (38.6%)	2980 (38.5%)	2985 (38.6%)
Unknown	856 (5.5%)	427 (5.5%)	429 (5.5%)	428 (5.5%)	428 (5.5%)
Vascular risk score*					
Low	6264 (40.5%)	3128 (40.4%)	3136 (40.5%)	3144 (40.6%)	3120 (40.3%)
Medium	6548 (42.3%)	3294 (42.6%)	3254 (42.0%)	3269 (42.2%)	3279 (42.4%)
High	2668 (17.2%)	1318 (17.0%)	1350 (17.4%)	1327 (17.1%)	1341 (17.3%)
Place of residence					
England	13960 (90.2%)	6990 (90.3%)	6970 (90.1%)	6939 (89.7%)	7021 (90.7%)
Other UK	1520 (9.8%)	750 (9.7%)	770 (9.9%)	801 (10.3%)	719 (9.3%)

Data are number of participants (%), mean (standard deviation), and median (interquartile range). *We categorised the predicted 5-year risk of serious vascular event without the use of aspirin or fatty acids as follows: low risk as less than 5%, moderate risk as 5% to less than 10%, and high risk as 10% or more.

© 2021 Harper C et al. *JAMA Network Open*.

eTable 3. Agreement of Routine Data vs Adjudicated Direct Follow-up for Any Serious Vascular Event Including Transient Ischemic Attack, by Subgroups

Subgroup	Outcome in both datasets	Outcome in routine data only	Outcome in adjudicated follow-up alone	No such outcome in either dataset	Sensitivity (95% CI)	Specificity (95% CI)	Kappa (95% CI)	Heterogeneity test*
**Age (years)**								
<63	316 (4%)	50 (1%)	136 (2%)	7128 (93%)	69.9% (65.7%-74.1%)	99.3% (99.1%-99.5%)	0.76 (0.73-0.79)	
≥63	693 (9%)	68 (1%)	256 (3%)	6833 (87%)	73.0% (70.2%-75.8%)	99.0% (98.8%-99.2%)	0.79 (0.77-0.81)	0.18
**Sex**								
Male	688 (7%)	76 (1%)	260 (3%)	8660 (89%)	72.6% (69.7%-75.4%)	99.1% (98.9%-99.3%)	0.78 (0.76-0.81)	
Female	321 (6%)	42 (1%)	132 (2%)	5301 (91%)	70.9% (66.7%-75.0%)	99.2% (99.0%-99.5%)	0.77 (0.74-0.80)	0.49
**Vascular risk score****								
Low	186 (3%)	29 (<1%)	117 (2%)	5932 (95%)	61.4% (55.9%-66.9%)	99.5% (99.3%-99.7%)	0.71 (0.66-0.75)	
Medium	458 (7%)	57 (1%)	164 (3%)	5869 (90%)	73.6% (70.2%-77.1%)	99.0% (98.8%-99.3%)	0.79 (0.76-0.81)	
High	365 (14%)	32 (1%)	111 (4%)	2160 (81%)	76.7% (72.9%-80.5%)	98.5% (98.0%-99.0%)	0.80 (0.77-0.84)	0.002
**Country**								
England	918 (7%)	106 (1%)	358 (3%)	12578 (90%)	71.9% (69.5%-74.4%)	99.2% (99.0%-99.3%)	0.78 (0.76-0.80)	
Other UK	91 (6%)	12 (1%)	34 (2%)	1383 (91%)	72.8% (65.0%-80.6%)	99.1% (98.7%-99.6%)	0.78 (0.72-0.84)	0.96
**All participants**	**1009 (7%)**	**118 (1%)**	**392 (3%)**	**13961 (90%)**	**72.0% (69.7%-74.4%)**	**99.2% (99.0%-99.3%)**	**0.78 (0.76-0.80)**	

Percentages in parentheses are % of total number of ASCEND participants. *Heterogeneity test compares kappa statistics between subgroups. **We categorised the predicted 5-year risk of serious vascular event without the use of aspirin or fatty acids as follows: low risk as less than 5%, moderate risk as 5% to less than 10%, and high risk as 10% or more. CI = Confidence intervals.

© 2021 Harper C et al. *JAMA Network Open*.

eTable 4. Agreement of Routine Data vs Adjudicated Direct Follow-up, by Diagnostic Position

Outcome by diagnostic position	Outcomes in both datasets	Outcomes in routine data only	Outcomes in adjudicated follow-up alone	No outcomes in either dataset	Sensitivity (95% CI)	Specificity (95% CI)	Kappa (95% CI)
Non-fatal myocardial infarction							
Any diagnostic position	304 (2%)	79 (1%)	82 (1%)	15015 (97%)	78.8% (74.7%-82.8%)	99.5% (99.4%-99.6%)	0.79 (0.75-0.82)
Primary diagnosis only	270 (2%)	46 (<1%)	116 (1%)	15048 (97%)	69.9% (65.4%-74.5%)	99.7% (99.6%-99.8%)	0.76 (0.73-0.80)
Non-fatal presumed ischaemic stroke							
Any diagnostic position	288 (2%)	65 (<1%)	143 (1%)	14984 (97%)	66.8% (62.4%-71.3%)	99.6% (99.5%-99.7%)	0.73 (0.69-0.76)
Primary diagnosis only	277 (2%)	53 (<1%)	154 (1%)	14996 (97%)	64.3% (59.7%-68.8%)	99.6% (99.6%-99.7%)	0.72 (0.68-0.76)
**Any serious vascular event excluding TIA**							
**Any diagnostic position**	**910 (6%)**	**116 (1%)**	**219 (1%)**	**14235 (92%)**	**80.6% (78.3%-82.9%)**	**99.2% (99.0%-99.3%)**	**0.83 (0.82-0.85)**
**Primary diagnosis only**	**868 (6%)**	**89 (1%)**	**261 (2%)**	**14262 (92%)**	**76.9% (74.4%-79.3%)**	**99.4% (99.3%-99.5%)**	**0.82 (0.80-0.84)**
Transient ischaemic attack							
Any diagnostic position	109 (1%)	30 (<1%)	256 (2%)	15085 (97%)	29.9% (25.2%-34.6%)	99.8% (99.7%-99.9%)	0.43 (0.36-0.49)
Primary diagnosis only	98 (1%)	20 (<1%)	267 (2%)	15095 (98%)	26.8% (22.3%-31.4%)	99.9% (99.8%-99.9%)	0.40 (0.33-0.47)
**Any serious vascular event including TIA**							
**Any diagnostic position**	**1009 (7%)**	**118 (1%)**	**392 (3%)**	**13961 (90%)**	**72.0% (69.7%-74.4%)**	**99.2% (99.0%-99.3%)**	**0.78 (0.76-0.80)**
**Primary diagnosis only**	**956 (6%)**	**88 (1%)**	**445 (3%)**	**13991 (90%)**	**68.2% (65.8%-70.7%)**	**99.4% (99.2%-99.5%)**	**0.76 (0.74-0.78)**

Percentages in parentheses are % of total number of ASCEND participants. CI = Confidence intervals. TIA = Transient ischaemic attack.

© 2021 Harper C et al. *JAMA Network Open*.

eTable 5. Comparison of Routine Data Event Date vs Adjudicated Direct Follow-up

**Outcome**	**Difference between event date in routine data and adjudicated follow-up**					
	**Exact match**	**1-7 days**	**8-30 days**	**31-90 days**	**91-180 days**	**>180 days**
Non-fatal myocardial infarction	223 (73%)	48 (16%)	11 (4%)	5 (2%)	2 (1%)	15 (5%)
Non-fatal presumed ischaemic stroke	216 (75%)	39 (14%)	9 (3%)	6 (2%)	3 (1%)	15 (5%)
Vascular death excluding intracranial haemorrhage	364 (>99%)	1 (<1%)	0 (0%)	0 (0%)	0 (0%)	0 (0%)
**Any serious vascular event excluding TIA**	**709 (78%)**	**93 (10%)**	**31 (3%)**	**18 (2%)**	**10 (1%)**	**49 (5%)**
Transient ischaemic attack	63 (58%)	14 (13%)	6 (6%)	6 (6%)	2 (2%)	18 (17%)
**Any serious vascular event including TIA**	**745 (74%)**	**112 (11%)**	**36 (4%)**	**23 (2%)**	**12 (1%)**	**81 (8%)**
Any arterial revascularisation	428 (62%)	201 (29%)	32 (5%)	8 (1%)	4 (1%)	12 (2%)
**Any serious vascular event or revascularisation**	**953 (68%)**	**235 (17%)**	**69 (5%)**	**37 (3%)**	**14 (1%)**	**96 (7%)**

Percentages in parentheses are % of total number of events where there was agreement. TIA = Transient ischaemic attack.

© 2021 Harper C et al. *JAMA Network Open*.

eTable 6. Information From Adjudicated Direct Follow-up for Events Only Considered to Be Serious Vascular Events in Routine Data

Information from adjudicated direct follow-up	Serious vascular events only in routine data
**Non-fatal myocardial infarction**	**54**
Reported but refuted during adjudication*	7
Unreported by participant	47
**Non-fatal presumed ischaemic stroke**	**40**
Reported but refuted during adjudication**	3
Unreported by participant	37
**Vascular death excluding intracranial haemorrhage**	**11**
Reported but refuted during adjudication†	11
Unreported	0
**Transient ischaemic attack**	**13**
Reported but refuted during adjudication‡	2
Unreported by participant	11
**Total**	**118**

*The 7 refuted non-fatal myocardial infarctions were adjudicated as cardiomyopathy (1 refuted event), heart failure (1), heart valve problem (1), atrial fibrillation (2), cardiac arrest (1), and angina (1). **The 3 refuted non-fatal presumed ischaemic strokes were adjudicated as haemorrhagic stroke (1), subdural haematoma (1), and prolapsed intervertebral disc (1). † The 11 refuted vascular deaths were adjudicated as cancer (6), benign neoplasm (1), intracranial haemorrhage (2), vascular dementia (1), and obesity (1). ‡ The 2 refuted transient ischaemic attacks were adjudicated as middle ear disease (1) and fit/convulsion unspecified (1).

© 2021 Harper C et al. *JAMA Network Open*.

eTable 7. Information From Routine Data for Events Only Considered to Be Serious Vascular Events in Adjudicated Direct Follow-up

Information from routine data	Serious vascular events only in adjudicated direct follow-up
**Non-fatal myocardial infarction**	**70**
Hospitalisation record within 90 days	
Other ischaemic heart disease code recorded*	44
No ischaemic heart disease code recorded	14
No hospitalisation record within 90 days	12
**Non-fatal presumed ischaemic stroke**	**93**
Hospitalisation record within 90 days	
Transient ischaemic attack	0
Other cerebrovascular disease code recorded**	10
No cerebrovascular disease code recorded	22
No hospitalisation record within 90 days	61
**Vascular death excluding intracranial haemorrhage**	**30**
Death record from English/Welsh/Scottish registries	
Vascular code recorded on the death certificate but not as underlying cause †	21
No vascular code recorded on the death certificate	0
No death record from English/Welsh/Scottish registries	9
**Transient ischaemic attack**	**199**
Hospitalisation record within 90 days	
Presumed ischaemic stroke code	0
Other cerebrovascular disease code recorded**	9
No cerebrovascular disease code recorded	60
No hospitalisation record within 90 days	130
**Total**	**392**

*ICD-10 codes for “other ischaemic heart disease” included I20 (angina pectoris), I23 (certain current complications following acute myocardial infarction), I24 (other acute ischaemic heart diseases), I25 (chronic ischaemic heart disease) in any diagnostic position. **ICD-10 codes for “other cerebrovascular disease” included G45.4 (transient global amnesia), G46 (vascular syndromes of brain in cerebrovascular diseases), I60-I69 (cerebrovascular diseases) excluding I63 (cerebral infarction) and I64 (stroke, not specified as haemorrhage or infarction) in any diagnostic position. †ICD-10 codes for “vascular death excluding intracranial haemorrhage” included I00:I52, I63:I99 (diseases of the circulatory system, excluding haemorrhagic stroke).

© 2021 Harper C et al. *JAMA Network Open*.

eTable 8. Agreement of Routine Data vs Adjudicated Direct Follow-up, Where Adjudicated Data Restricted to Fatal and Hospitalized Event Only

Outcome	Outcome in both datasets	Outcome in routine data only	Outcome in adjudicated follow-up alone	No such outcome in either dataset	Sensitivity (95% CI)	Specificity (95% CI)	Kappa (95% CI)
Non-fatal myocardial infarction	296 (2%)	87 (1%)	70 (<1%)	15027 (97%)	80.9% (76.8%-84.9%)	99.4% (99.3%-99.5%)	0.79 (0.75-0.82)
Non-fatal presumed ischaemic stroke	260 (2%)	93 (1%)	46 (<1%)	15081 (97%)	85.0% (81.0%-89.0%)	99.4% (99.3%-99.5%)	0.78 (0.75-0.82)
Vascular death excluding intracranial haemorrhage	365 (2%)	18 (<1%)	49 (<1%)	15048 (97%)	88.2% (85.1%-91.3%)	99.9% (99.8%-99.9%)	0.91 (0.89-0.93)
**Any serious vascular event excluding TIA**	**879 (6%)**	**147 (1%)**	**123 (1%)**	**14331 (93%)**	**87.7% (85.7%-89.8%)**	**99.0% (98.8%-99.1%)**	**0.86 (0.84-0.87)**
Transient ischaemic attack	71 (<1%)	68 (<1%)	49 (<1%)	15292 (99%)	59.2% (50.4%-68.0%)	99.6% (99.5%-99.7%)	0.54 (0.46-0.63)
**Any serious vascular event including TIA**	**950 (6%)**	**177 (1%)**	**149 (1%)**	**14204 (92%)**	**86.4% (84.4%-88.5%)**	**98.8% (98.6%-98.9%)**	**0.84 (0.83-0.86)**
Any arterial revascularisation	685 (4%)	51 (<1%)	39 (<1%)	14705 (95%)	94.6% (93.0%-96.3%)	99.7% (99.6%-99.7%)	0.94 (0.92-0.95)
**Any serious vascular event or revascularisation**	**1361 (9%)**	**170 (1%)**	**138 (1%)**	**13811 (89%)**	**90.8% (89.3%-92.3%)**	**98.8% (98.6%-99.0%)**	**0.89 (0.87-0.90)**

Percentages in parentheses are % of total number of ASCEND participants. Hospitalised events defined as any event where adjudicated follow-up reported that the participant was admitted to hospital. CI = Confidence intervals. TIA = Transient ischaemic attack.

© 2021 Harper C et al. *JAMA Network Open*.

eTable 9. Comparison of Estimated Treatment Effect Sizes Between Adjudicated Follow-up and Routine Data for the Aspirin and ω-3 Fatty Acids Randomized Comparisons

	Rate ratios (95% confidence interval)					
	Aspirin vs placebo			ω-3 fatty acids vs placebo		
**Outcome**	**Adjudicated follow-up**	**Routine data**	**Difference in point estimate***	**Adjudicated follow-up**	**Routine data**	**Difference in point estimate***
Non-fatal myocardial infarction	0.98 (0.80-1.19)	1.00 (0.82-1.22)	+0.03 (-0.11, +0.16)	0.93 (0.76-1.14)	0.96 (0.79-1.18)	+0.03 (-0.09, +0.17)
Non-fatal presumed ischaemic stroke	0.88 (0.73-1.06)	0.82 (0.66-1.01)	-0.06 (-0.18, +0.07)	1.01 (0.84-1.22)	1.02 (0.82-1.25)	+0.00 (-0.15, +0.16)
Vascular death excluding intracranial haemorrhage	0.91 (0.75-1.10)	0.86 (0.70-1.05)	-0.05 (-0.12, +0.02)	0.81 (0.67-0.99)	0.76 (0.62-0.92)	-0.06 (-0.13, +0.01)
**Any serious vascular event excluding TIA**	**0.92 (0.82-1.03)**	**0.91 (0.80-1.02)**	**-0.01 (-0.07, +0.05)**	**0.93 (0.83-1.05)**	**0.92 (0.81-1.04)**	**-0.01 (-0.08, +0.06)**
Transient ischaemic attack	0.85 (0.69-1.04)	0.96 (0.69-1.33)	+0.11 (-0.14, +0.44)	1.03 (0.84-1.26)	1.14 (0.81-1.58)	+0.11 (-0.18, +0.52)
**Any serious vascular event including TIA**	**0.88 (0.79-0.97)**	**0.91 (0.81-1.02)**	**+0.03 (-0.04, +0.10)**	**0.97 (0.87-1.08)**	**0.94 (0.84-1.06)**	**-0.03 (-0.10, +0.05)**
Any arterial revascularisation	0.88 (0.76-1.02)	0.89 (0.77-1.02)	+0.01 (-0.04, +0.05)	1.04 (0.90-1.20)	1.06 (0.92-1.22)	+0.02 (-0.04, +0.08)
**Any serious vascular event or revascularisation**	**0.88 (0.80-0.97)**	**0.90 (0.81-0.99)**	**+0.02 (-0.03, +0.07)**	**1.00 (0.91-1.09)**	**0.99 (0.89-1.09)**	**-0.01 (-0.07, +0.05)**

Log-rank methods were used to calculate the rate ratio and 95% confidence intervals. *Bootstrap methods were used to calculate the 95% confidence intervals for the difference in rate ratios between adjudicated follow-up and routine data. These methods used 1000 resamplings, with replacement, where the difference in point estimate was recalculated in each bootstrap sample. TIA = Transient ischaemic attack.

© 2021 Harper C et al. *JAMA Network Open*.

eFigure 1. Methods of Follow-up in the ASCEND Trial

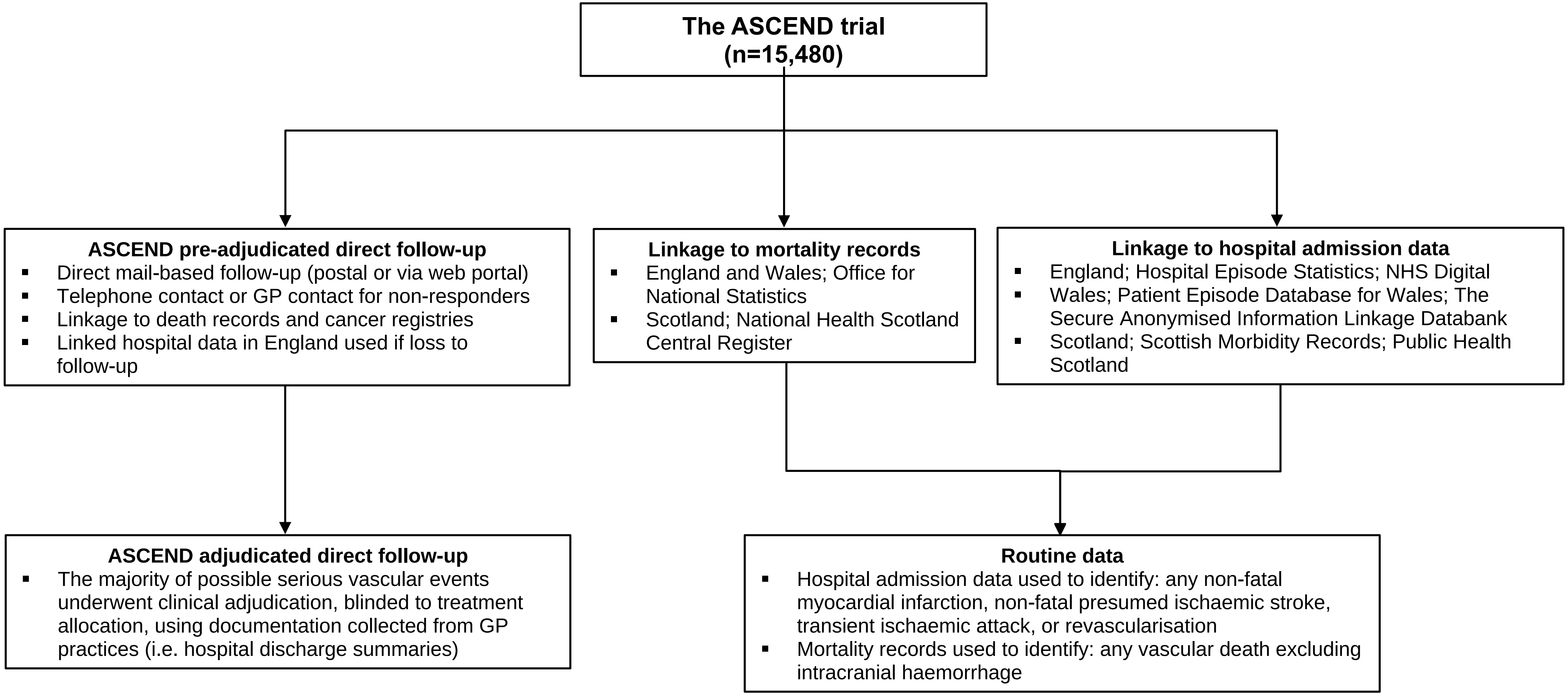

© 2021 Harper C et al. *JAMA Network Open*.

eFigure 2. Association of Allocation to Aspirin vs Matching Placebo and to ω-3 Fatty Acids vs Matching Placebos With Any Serious Vascular Event or Revascularization Using Routine Data Follow-up, Where Adjudicated Data Restricted to Fatal and Hospitalized Event Only

Legend: Adjudicated direct follow-up (black square); Routine data follow-up (red square)

Outcome	Follow-up	Aspirin (n=7,740)	Placebo (n=7,740)	Rate ratio (95% CI)	Fatty acids (n=7,740)	Placebo (n=7,740)	Rate ratio (95% CI)
Non-fatal myocardial infarction	Adjudicated direct	182 (2.4%)	184 (2.4%)	0.99 (0.80-1.21)	175 (2.3%)	191 (2.5%)	0.92 (0.75-1.12)
	Routine data	192 (2.5%)	191 (2.5%)	1.00 (0.82-1.22)	188 (2.4%)	195 (2.5%)	0.96 (0.79-1.18)
Non-fatal presumed ischaemic stroke	Adjudicated direct	139 (1.8%)	167 (2.2%)	0.83 (0.66-1.04)	153 (2.0%)	153 (2.0%)	1.00 (0.80-1.25)
	Routine data	159 (2.1%)	194 (2.5%)	0.82 (0.66-1.01)	178 (2.3%)	175 (2.3%)	1.02 (0.82-1.25)
Vascular death excluding intracranial haemorrhage	Adjudicated direct	197 (2.5%)	217 (2.8%)	0.91 (0.75-1.10)	186 (2.4%)	228 (2.9%)	0.81 (0.67-0.99)
	Routine data	177 (2.3%)	206 (2.7%)	0.86 (0.70-1.05)	165 (2.1%)	218 (2.8%)	0.76 (0.62-0.92)
**Any serious vascular event excluding TIA**	Adjudicated direct	**477 (6.2%)**	**525 (6.8%)**	**0.90 (0.80-1.02)**	**475 (6.1%)**	**527 (6.8%)**	**0.90 (0.80-1.02)**
	Routine data	**489 (6.3%)**	**537 (6.9%)**	**0.91 (0.80-1.02)**	**491 (6.3%)**	**535 (6.9%)**	**0.92 (0.81-1.04)**
Transient ischaemic attack	Adjudicated direct	61 (0.8%)	59 (0.8%)	1.03 (0.72-1.48)	68 (0.9%)	52 (0.7%)	1.31 (0.91-1.87)
	Routine data	68 (0.9%)	71 (0.9%)	0.96 (0.69-1.33)	74 (1.0%)	65 (0.8%)	1.14 (0.81-1.58)
**Any serious vascular event including TIA**	Adjudicated direct	**526 (6.8%)**	**573 (7.4%)**	**0.91 (0.81-1.03)**	**536 (6.9%)**	**563 (7.3%)**	**0.95 (0.85-1.07)**
	Routine data	**537 (6.9%)**	**590 (7.6%)**	**0.91 (0.81-1.02)**	**547 (7.1%)**	**580 (7.5%)**	**0.94 (0.84-1.06)**
Any arterial revascularisation	Adjudicated direct	340 (4.4%)	384 (5.0%)	0.88 (0.76-1.02)	368 (4.8%)	356 (4.6%)	1.04 (0.90-1.20)
	Routine data	347 (4.5%)	389 (5.0%)	0.89 (0.77-1.02)	378 (4.9%)	358 (4.6%)	1.06 (0.92-1.22)
**Any serious vascular event or revascularisation**	Adjudicated direct	**713 (9.2%)**	**786 (10.2%)**	**0.90 (0.81-1.00)**	**743 (9.6%)**	**756 (9.8%)**	**0.99 (0.89-1.09)**
	Routine data	**726 (9.4%)**	**805 (10.4%)**	**0.90 (0.81-0.99)**	**761 (9.8%)**	**770 (9.9%)**	**0.99 (0.89-1.09)**

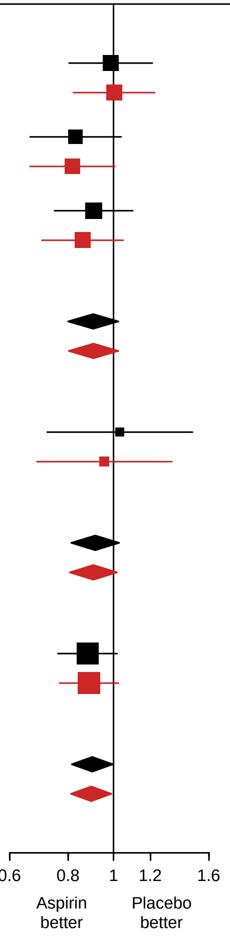

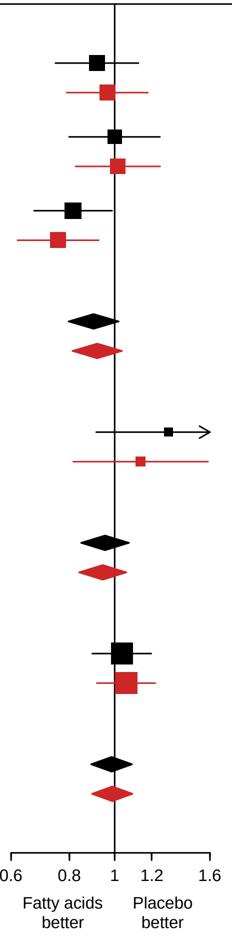

Log-rank methods were used to calculate the rate ratio and 95% confidence intervals. Hospitalised events defined as any event where adjudicated follow-up reported that the participant was admitted to hospital. CI = Confidence intervals. TIA = Transient ischaemic attack.

© 2021 Harper C et al. *JAMA Network Open*.
