# Supplementary material for: Comparison of the Accuracy and Completeness of Records of Serious Vascular Events in Routinely Collected Data vs Clinical Trial–Adjudicated Direct Follow-up Data in the UK: Secondary Analysis of the ASCEND Randomized Clinical Trial
Source: JAMA Netw Open. 2021 Dec 28;4(12):e2139748. doi: 10.1001/jamanetworkopen.2021.39748 (PMC8715347; doi:10.1001/jamanetworkopen.2021.39748)
Supplement: Supplement 3. — Data Sharing Statement [file jamanetwopen-e2139748-s003.pdf]

Data Sharing Statement

Harper. Comparison of the Accuracy and Completeness of Records of Serious Vascular Events in Routinely Collected Data vs Clinical Trial-Adjudicated Direct Follow-up Data in the UK. *JAMA Netw Open*. Published December 28, 2021. doi:10.1001/jamanetworkopen.2021.39748

Data
Data available: No

Additional Information
Explanation for why data not available: Post-trial follow-up of the ASCEND cohort is on-going using the routinely collected data, with planned analyses 5 and 10 years after the end of the scheduled treatment period. Data sharing will be considered in line with the Nuffield Department of Population Health, University of Oxford, Data Access and Sharing Policy available at https://www.ndph.ox.ac.uk/about/data-access-policy.
